# Defect Tolerant Quantum Cutting in Mechanosynthesized Ytterbium-Doped Cesium Lead Chloride Perovskites

**DOI:** 10.1021/acs.chemmater.5c02211

**Published:** 2026-01-12

**Authors:** Thiago I. Rubio, Claudia E. Avalos

**Affiliations:** Department of Chemistry, 5894 New York University, New York, New York 10003, United States

## Abstract

Ytterbium doped cesium lead halide materials exhibit a property known as quantum cutting which allows for greater than 100% photoluminescent quantum yields (PLQYs). The local atomic structure of the defects responsible for these properties and the effectiveness of the doping for producing the desired PLQYs is not readily discerned using techniques requiring long-range order. In this work we prepared 2.5, 5, 10, and 20% Yb^3+^ doped CsPbCl_3_ powders using mechanosynthesis under distinct stoichiometric ratio conditions and characterized the defect incorporation and its effects on local atomic disorder using solid-state nuclear magnetic resonance (SSNMR) spectroscopy. We then correlate our observations to the observed PLQYs for each of the prepared samples. All samples prepared were found to be in an orthorhombic phase and no lattice shrinking was observed upon increased Yb^3+^ doping. An increase in doping concentrations was accompanied by a decrease in ^133^Cs NMR spin–lattice relaxation times *T*_1_ consistent with a paramagnetic relaxation enhancement effect induced by Yb^3+^ incorporation into the perovskite lattice. Through a comparison of synthesis methods, PLQY and NMR *T*_1_ parameters we found that incorporated defects favorable for PLQY in mechanosynthesized samples are more likely to form in the presence of excess lead and excess chloride ions. The maximum PLQY values obtained for each set of samples correlated with *T*_1_ parameters in the range of 13 to 35 s. In addition, we found that the observed PLQY in 5% doped samples was optimized after 1 to 2 h of interval grinding in stainless steel jars. Further grinding beyond 2 h led to a reduction in particle size below 1 μm as well as a reduction in PLQY and spin relaxation times.

## Graphical abstract

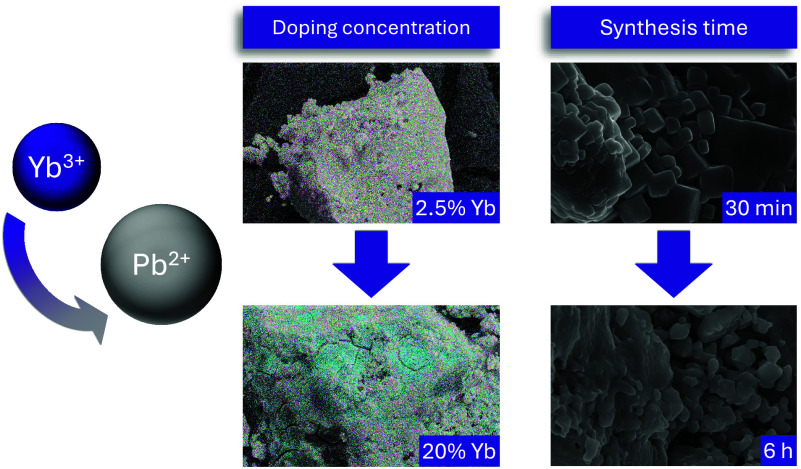

## 1 Introduction

In the ever-growing field of photovoltaics, inorganic halide perovskites have emerged as competitive alternatives to silicon-based devices.^1^ Their versatility arises from the ease of tunability of their optical properties by varying their composition as well as their high tolerance to incorporation of doping agents. In fact, the properties of halide perovskites are known to be tolerant to the incorporation of impurities.^2,3^ This means that even under fairly relaxed synthesis conditions, for example at room temperature and in air, the defect densities are small. Such densities are comparable to what is achieved under significantly more rigorous conditions for other classes of optoelectronic semiconductors.^4^ The formation of defects in halide perovskites is inherent in growth conditions and synthesis methods. Point defects are the most common type and encompass substitutionals, antisite occupations, vacancies and interstitials. The formation of grain boundaries is an example of a defect of higher dimension, with other examples including dislocations and clustering or precipitate formation. Introducing additional species into the crystal lattice without causing significant disruptions can allow distinct electronic processes to occur, thus expanding the range of uses of these materials.^5–7^ One such photophysical process mediated by defects is known as quantum cutting. Quantum cutting (QC) is defined as the emission of more than one low-energy photon following the absorption of one high-energy photon. Halide perovskites, when doped with lanthanide ions, are capable of exhibiting this process. One example is that of cesium lead halide perovskites (CsPbX_3_) doped with Yb^3+^ which have been shown to exhibit photoluminescent quantum yields (PLQY) as high as 190% in nanocrystalline samples.^8^ This type of material sees a potential application as a downconversion layer in a solar cell – where the generated photons can then be absorbed by another photoactive layer that will promote charge separation, thus increasing the overall efficiency of the device.^9,10^ The quantum cutting process in CsPbX_3_ involves the absorption of radiation from the valence band to the conduction band, followed by nonradiative energy transfer between shallow defects below and close in energy (on the order of ∼*k*_B_T) to the conduction band.^9,11,12^ A lead vacancy, V_Pb_, has been proposed to mediate the rapid energy transfer to two Yb^3+^ neighbors^9^ which will, in turn, emit photons in the near-infrared region, corresponding to the ^2^F_5/2_ → ^2^F_7/2_transition of Yb^3+^, shown in Figure 1. Based on photoluminescence measurements as well as its low energy of formation, an Yb^3+^-V_Pb_-Yb^3+^ complex has been proposed as being the relevant defect to facilitate quantum cutting in these materials,^11^ where two Yb^3+^ atoms replace three Pb^2+^ atoms, leaving a vacancy site, V_Pb_, to maintain charge neutrality.^9,13,14^ It has also been proposed that the mechanism responsible for luminescence may depend on the average grain size of the material.^15,16^ For example in 9.9% Yb:CsPbCl_3_ formed via solvent assisted reagent mixing in an agate mortar and high temperature annealing,^15^ QC is proposed to be mediated via a generated free exciton before transferring to the relevant V_Pb_ defect. In this case^15^ the measurements are done on micron-sized crystals found in the orthorhombic phase rather than on nanocrystalline forms which are found in higher symmetry phases (tetragonal and cubic) at room temperature. The smaller grain sizes of the nanocrystalline forms are also associated with a greater prevalence of surface defects and grain boundaries.

**Figure 1:**
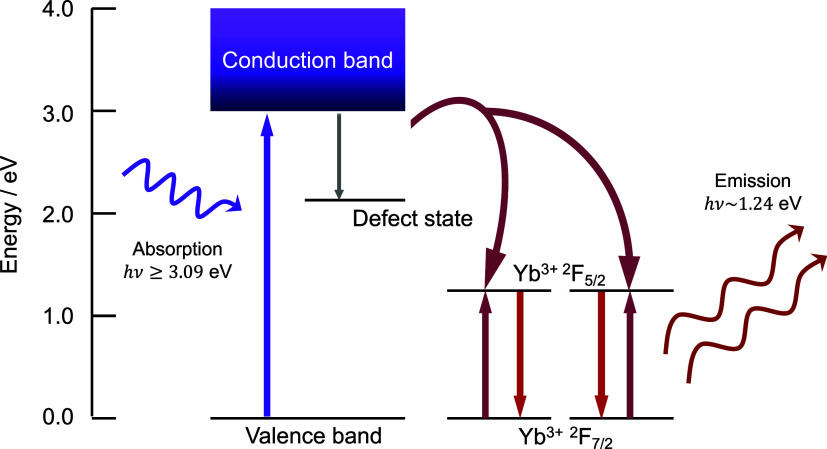
Energy diagram illustrating the quantum cutting effect in Yb^3+^ doped CsPbCl_3_. The band gap energy of CsPbCl_3_ as well as the IR emission energy has previously been reported and these values are reproduced in the figure shown here.^23,24^

Quantum cutting has been demonstrated in cesium lead halide perovskites synthesized using several different methods, such as physical vapor deposition,^17^ thin-film deposition via spin coating,^10^ and hot-injection^11,18,19^ with respective PLQYs for Yb^3+^ doped CsPbCl^_3_^ shown in Table 1. The common point among the higher PLQY values is their synthesis method, which yields the perovskite material in its nanocrystalline form. Perovskite nanocrystals are found in higher symmetry phases (tetragonal and cubic), unlike their microcrystalline counterparts (orthorhombic), because the size reduction to nanoscale dimensions promotes a change in the particle volume and surface Gibbs free energy contributions, thus rendering the aforementioned phases stable at room temperatures.^20–22^ Therefore, in addition to the size of the particle, the phase may also play an important role in the relevant mechanism leading to a large PLQY.

**Table 1:** Photoluminescence Quantum Yields for Yb-Doped CsPbCl_3_ in Different Works

work	synthesis method	crystal phase	Yb^3+^ conc. (mol %)	PLQY (%)
^12^	Hot injection (nanocrystals)	Tetragonal	7.2–9.1	143
 ^11^	Hot injection (nanocrystals)	-	5.2	170
 ^29^	Hot injection (nanocrystals)	Tetragonal	2.0	128
 ^26^	Hot injection (nanocrystals)	-	4.5	127
 ^19^	Modified hot injection (nanocrystals)	Cubic	7.0	110
 ^30^	Aerosol-assisted chemical vapor deposition (film)	-	50	68
 ^31^	Bridgman (single crystal)	-	2.0	86
 ^32^	Physical vapor deposition	Orthorhombic	5.0	70
 ^15^	Mechanosynthesis (mortar and pestle, liquid assisted)	Orthorhombic	9.0	76
This work	Mechanosynthesis	Orthorhombic	5.0	78

However, although these materials are well studied, there is still a debate on how the relevant defect incorporation occurs.^8,9,11,12,25,26^ Experimental techniques that support the Yb-V_Pb_-Yb type of defect site include extended X-ray absorption fine structure and X-ray pair distribution function analysis.^27^ However, atomic-scale imaging results show evidence of Yb^3+^ occupying interstitial sites as well as substitutional sites in the lattice.^28^

Identifying incorporation of dopants in materials can often be challenging given the low concentrations involved and the absence of defect contribution to changes in bulk order. One method that has been successfully used to probe dopant incorporation in halide perovskites is solid-state nuclear magnetic resonance (SSNMR) spectroscopy.^33^ In general, the incorporation of paramagnetic dopants into diamagnetic materials leads to changes in the spin–lattice relaxation time, *T*_1_, of spin active nuclei within the lattice. This effect is known as paramagnetic relaxation enhancement (PRE) and arises due to the hyperfine interaction between unpaired electrons in the sample and neighboring nuclei. This effect has been observed in paramagnetically doped lead halide perovskites, including lanthanide doped CsPbCl_3_ at fixed dopant concentrations.^34^ In addition to changes in the observed spin–lattice relaxation time of ^133^Cs, in some cases doping of Yb^3+^ in CsPbCl_3_ leads to the formation of additional cesium sites as evidenced by the appearance of additional ^133^Cs resonances in solid-state NMR spectra of doped samples. Both of these results support the incorporation of Yb^3+^ into CsPbCl_3_ as well as the introduction of local disorder upon Yb^3+^ incorporation.

However, questions remain as to how Yb^3+^ may be incorporated in CsPbCl_3_ in order to maximize the defects associated with increased quantum yield as well as to how the grain size is related to the observed PLQY. To this end, we prepared a series of samples using mechanochemical synthesis with varied reagent ratios, dopant concentrations and ball-milling times. We note that ball-milling is a single step solvent-free method, in which reactions take place due to energy provided to the starting materials by the mechanical shock of the particles between themselves, with the walls of the grinding jar, and the ball that is added to the vessel.^35–37^ Increasing milling time has the effect of reducing particle sizes and introducing additional defects such as vacancies, dislocations and grain boundaries, as has been previously investigated to enhance properties in other materials.^38–41^

In this work we use a combination of solid-state NMR spectroscopy, photoluminescence measurements, and powder X-ray diffraction to assess the relationship between local atomic structure, spin–lattice relaxation times, photoluminescence quantum yields and synthesis approach. We find that the QC mechanism is highly defect tolerant and that by varying the milling times and reagent ratios we successfully increased the number of relevant defect sites contributing to QC for a given Yb^3+^ concentration. We also identify a relationship between the range of spin–lattice relaxation times observed and the maximum PLQY measured.

## 2 Experimental Methods

### 2.1 Synthesis

All starting materials were obtained from Sigma-Aldrich: lead chloride (PbCl_2_, 99.999%), cesium chloride (CsCl, 99.9%), and ytterbium chloride (YbCl_3_, 99.99%). A 1:1 molar ratio of CsCl:PbCl_2_ was used in all compositions except where otherwise noted, with a total of 2.5 mmol synthesized each time. The starting materials were added to an agate (or stainless steel) jar, along with a 10 mm agate (or stainless steel) ball (sealed under N_2_ atmosphere). The mixed powders were then ball-milled in a Retsch MM400 mixer mill at 30 Hz for a period of 30 min. In the case of prolonged milling times, the ball-milling time was varied from 30 min to 6 h, with 5 min rest time at every hour. All products were then annealed under air at 450 °C for 60 min in a tube furnace. A reaction scheme is included in the Supporting Information.

### 2.2 X-ray Diffraction

Powder X-ray diffraction patterns were recorded on all the synthesized powders using a Bruker AXS D8 Discover diffractometer equipped with a General Area Detector Diffraction System (GADDS) and a high intensity ceramic seal tube emitting Cu Kα X-rays (1.5405 Å). The diffractograms were integrated between 2θ values of 10 and 50°. The samples were placed on a sample holder provided with the instrument and measurements were collected in reflection mode.

### 2.3 Scanning Electron Microscopy and Energy-Dispersive X-ray Spectroscopy

SEM images were acquired on a Carl Zeiss MERLIN field emission scanning electron microscope using a combination of two secondary electron detectors–Everhart-Thornley and annular types. EDX maps were obtained using an Ultim Max Energy Dispersive Spectroscopy detector from Oxford. All images and measurements were taken at an accelerating voltage of 15 kV. For the analysis, the synthesized powders were spread on carbon tape and coated with 5 nm gold through sputtering using a EMS 150R ES sputter coater.

### 2.4 Solid-State Nuclear Magnetic Resonance Spectroscopy

^133^Cs and ^207^Pb MAS NMR spectra were collected at 18.79 T (800 MHz ^1^H) on a Bruker Avance NEO NMR spectrometer, using a PhoenixNMR 1.6 mm CPMAS solid-state probe. The samples were packed in 1.6 mm zirconia pencil rotors. All measurements were taken between 240 and 260 K with a 22 kHz spin rate except where noted. The solid-state NMR spectra were collected using a standard echo sequence (90°−τ–180°–acquire, τ being the interpulse time delay).^42^ 90° pulses were calibrated and set as 6 μs for ^133^Cs and 7 μs for ^207^Pb at 50 W pulse power. Eight accumulations were collected for ^133^Cs spectra, and 200 accumulations were collected for ^207^Pb spectra, except where otherwise noted. Recycle delays were optimized as 1.3·*T*_1_, except where otherwise noted, after determination of spin–lattice relaxation time constants via an echo saturation recovery pulse sequence. Static ^207^Pb NMR spectra were collected at 17.62 T (750 MHz ^1^H) on a Bruker Avance NEO NMR spectrometer, using a 4 mm CPMAS solid-state probe. The samples were packed in 4 mm rotors, and all measurements were taken at 295 K. A full echo was collected, with 90° pulses calibrated and set as 4 μs at 800 W pulse power. 30,000 accumulations were collected, with a recycle delay of 1.3·*T*_1_. Solid CsI was used as a secondary reference, 271.05 ppm,^43^ for ^133^Cs spectra. Pb­(NO_3_)_2_ (−3490 ppm at 295 K) was used as the secondary reference for ^207^Pb spectra.^44^ Static solid-state spectra were fitted using the Dmfit software package.^45^ Spectral simulations were performed using WSolids1 software package.^46^

### 2.5 Photoluminescence Spectroscopy and Photoluminescence Quantum Yield

Photoluminescence spectra were collected using a Horiba QuantaMaster-8075–21 spectrophotometer at room temperature. The powders were added to a Spectralon sample holder adapter that is specific for measuring powders and the samples were excited at 360 nm with double monochromator-filtered emission from a Xe-arc lamp. A PMT was used for detection in the visible range, whereas a liquid nitrogen-cooled InGaAs photodetector was used for detection in the near-infrared range. An integration sphere was used in all measurements to allow for accurate PLQY determination on powder samples, and the lamp power was measured using a ThorLabs PM100A power meter. Information about how PLQYs were calculated is included in the Supporting Information. To check for precision of the method, triplicate measurements were performed on one of the sets of samples (later labeled series G2). The error bars reported represent the standard deviation of the average value of the PLQY.

## 3 Results

### 3.1 Effects of Yb^3+^ Doping on Properties of Mechanosynthesized CsPbCl_3_

In order to investigate the PLQYs of mechanosynthesized Yb^3+^ doped CsPbCl_3_, we prepared five samples with YbCl_3_ concentrations varying between 2.5 and 20 mol % using an agate grinding jar and 10 mm agate ball. The ytterbium salt was added to an equimolar mixture of PbCl_2_ and CsCl. These Yb^3+^ doped CsPbCl_3_ samples were mechanosynthesized in agate jars and prepared with excess chloride. An excess of chloride, Cl^–^ was used in this synthesis as it has been shown to reduce the energy of formation of a Pb–Cl vacancy pair and to promote the incorporation of Mn^2+^ into CsPbCl_3_ via reduced energy of formation of an incorporated Mn–Cl pair.^47,48^ Although the mechanism of incorporation is likely distinct due to the difference in charge of the incorporated ion (here we consider Yb^3+^ incorporation as opposed to Mn^2+^) the reduced energy of formation of the Pb–Cl vacancy pair should still apply to our samples and could facilitate the formation of Yb–Cl pairs.^47^ This series of samples is labeled series A.

#### 3.1.1 X-ray Diffraction

The phase purity of these samples was characterized using powder X-ray diffraction as shown in Figure 2. XRD patterns indicate that one phase was obtained for low doping concentrations, consistent with the orthorhombic structure (space group *Pnma*),^49^ which has previously been found to be the primary phase measured at room temperature for powder samples synthesized via a solvent-assisted grinding method.^15^ Above 10% of Yb^3+^, a secondary phase is observed at 28.5° (shown in detail in Figure S1b) which does not correspond to the starting materials or their simple oxides (e.g., PbO, YbO, etc.). We suspect that this phase, which is present in small amounts, may correspond to a more complex oxide phase.

**Figure 2:**
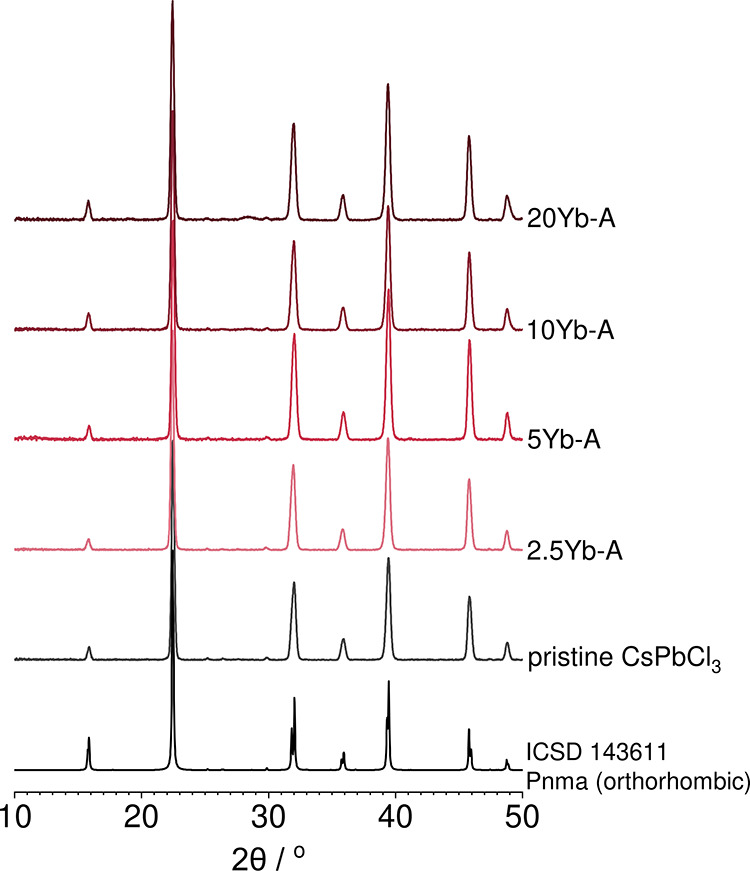
X-ray diffraction patterns of the synthesized perovskite samples (series A). From bottom to top, the patterns correspond to simulated pattern signals from the CsPbCl^_3_^ orthorhombic reference, the pristine mechanosynthesized CsPbCl_3_ sample (0 mol % Yb, 30 min/30 Hz milling), and the doped samples with 2.5, 5, 10, and 20 mol % of added ytterbium cations.^49^

No appreciable shifts in the XRD data were observed between the undoped and doped samples, even up to 20% doping. This indicates that even at high doping concentrations of Yb^3+^, the structure of the perovskite host is retained. Although other studies reported shrinking of the lattice structure with the introduction of ytterbium cations in CsPbCl_3_ prepared in a nanocrystalline form via solution methods,^48,50^ this has not been observed for thin film samples prepared via solution processing and powders produced via solid-state synthesis.^15,51^ In the case of nanocrystalline CsPbCl_3_,^48^ a peak shift of 0.42° was reported for the peak assigned to the (110) planes (22.5°), whereas in our mechanosynthesized samples a shift of <0.2° is observed between the pristine form and the highest doped sample, as seen in Figure S1a.

#### 3.1.2 Scanning Electron Microscopy and Energy-Dispersive X-ray Spectroscopy

SEM images were collected in order to characterize the morphology of the doped samples as well as their elemental composition. A change in morphology is observed moving from 0% doping to 20% doping (Figure S2). The pristine and 2.5% samples have a smooth surface with divots present in larger particle components which may arise as part of thermal annealing. At 10% doping grain boundaries are less well-defined and at 15% the smaller particles appear to start aggregating. Cracks are observed in the 20% sample which may be consistent with corroded grain boundaries or localized lattice shrinking. In addition we observe smaller particles with a powdery appearance near the formed cracks. These cracks appear in regions where ytterbium is aggregated as shown in energy-dispersive X-ray maps. Energy-dispersive X-ray spectroscopy (EDX) maps provide information on the spatial distribution of each element across the surface of the samples, which can be used to evaluate whether precipitates of any element were formed. Figure 3 shows the EDX maps for the case of 20% and 10% doping.

**Figure 3:**
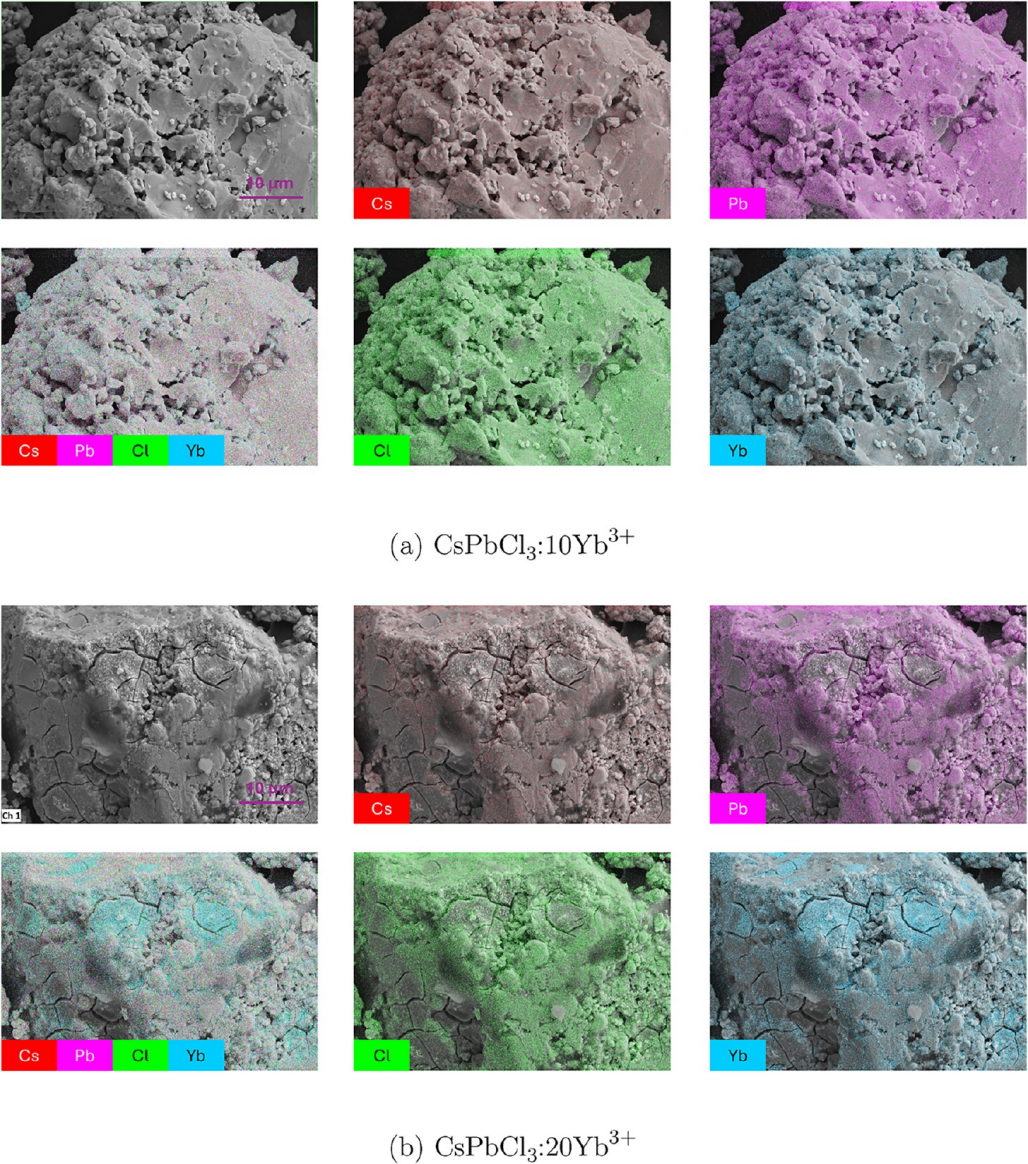
SEM and EDX maps of (a) CsPbCl_3_:10Yb^3+^ and (b) CsPbCl_3_:20Yb^3+^. For both figures, on top left: SEM image; the other five images are colored overlays of EDX mappings for each of the elements in the sample: red for Cs, pink for Pb, green for Cl and blue for Yb; a composed map of all elements is shown on the bottom left image, where areas containing a higher concentration of Yb atoms can be seen.

As can be seen, at a concentration of 20 mol % of Yb^3+^ (Figure 3b), ytterbium cations precipitate into clusters. This is evident in the EDX map where all elements are overlaid. In the regions where Yb is concentrated, the blue color is more apparent. It can also be noted in the individual element maps that there is a lack of coloration for Cs, Pb and Cl in the areas where Yb is clustering. This effect is not as pronounced at a concentration of 10 mol % (Figure 3a). At lower concentrations of Yb^3+^, a more uniform distribution of all elements is observed (Figure S3). No precipitation of ytterbium in the lattice was observed in samples at 2.5 and 5% Yb^3+^ doping.

#### 3.1.3 ^133^Cs Solid-State Nuclear Magnetic Resonance Spectroscopy

As has been established previously,^34^ ^133^Cs can serve as a reporter nucleus to assess lanthanide incorporation. We measured ^133^Cs NMR spectra and spin–lattice relaxation times for mechanosynthesized samples prepared at different ytterbium doping concentrations. All spectra shown in Figure 4a were taken at a fixed temperature setting of 240 ± 5 K. For the case of the pristine sample, a single cesium site is observed with a chemical shift of 83.4 ppm, consistent with previous observations^36^ when temperature dependence is taken into account. For example, the difference in chemical shift values observed in this study as compared to literature values are consistent with ^133^Cs chemical shifts in perovskites being temperature-dependent, as shown for mechanosynthesized CsPbCl_3_ in Figure S4, with a temperature dependence of −0.20 ppm/K, where we observe a decrease in the chemical shift as the temperature of the sample increases. Additionally, as the amount of Yb^3+^ in the composition increases, the ^133^Cs resonance broadens linearly with respect to Yb^3+^ concentration as shown in Figures 4b and S6c.

**Figure 4:**
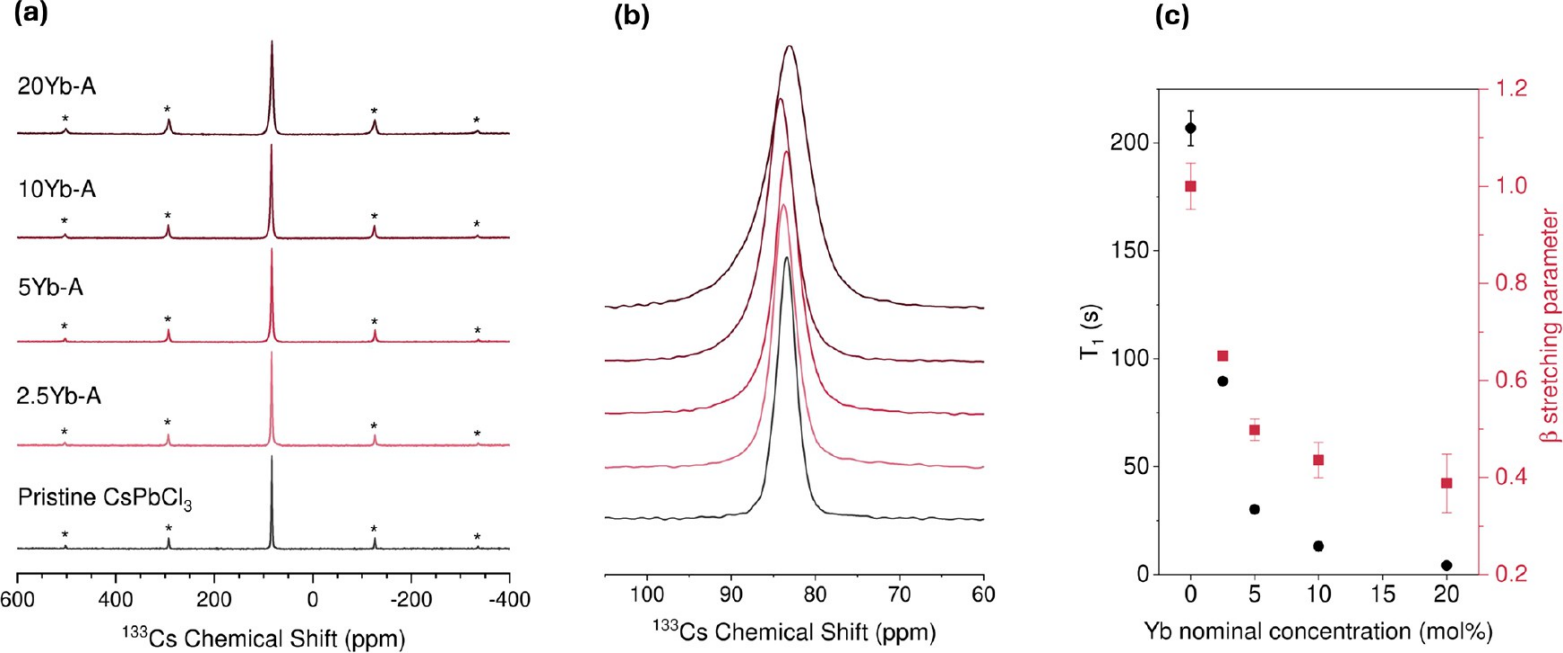
(a) ^133^Cs solid-state NMR spectra (18.8 T, 240 K, 22 kHz spin rate) for the mechanosynthesized perovskite samples with different concentrations of Yb^3+^ (series A). (b) Close-up of the central feature. A broadening of the signal with increasing amounts of Yb^3+^ added to the composition can be observed. (c) *T*_1_ relaxation time-constants (black) and β stretching parameter (red) for ^133^Cs nuclei in the perovskite samples of series A.

In order to further investigate the incorporation of Yb^3+^ into the perovskite lattice, saturation recovery experiments were conducted to extract the spin–lattice relaxation time constant (*T*_1_) parameter. The incorporation of Yb^3+^ into the CsPbCl_3_ lattice leads to a PRE effect at the ^133^Cs site. In the absence of spin diffusion, the magnitude of this effect scales as 1/*r* ^6^ where *r* is the distance between two magnetic centers^52,53^ and the spin relaxation profile may be fit using a stretched exponential as shown in eq 1.

$$I\left(\tau \right)=I_{0}[1-\exp\left(-{\left(\frac{\tau }{T_{1}}\right)}^{\beta }\right)],$$ 1

where *T*_1_ is the spin–lattice relaxation time and β is a parameter that is related to a distribution of relaxation times. A β parameter closer to 1 has been associated with a homogeneous magnetic environment around the relevant nucleus while as the value of β approaches 0.5, a larger distribution of environments is typically inferred due to the presence of paramagnetic species. A β value of 0.5 is typically interpreted as consistent with a random distribution of the paramagnetic species around the relevant nuclear site.^54–56^ Figure 4c shows *T*_1_ obtained using a stretched exponential fit and the respective β parameter for each of the samples. We observed a decrease in the spin–lattice relaxation time of ^133^Cs with an increasing amount of ytterbium in the composition. For the undoped sample, the β parameter is found to be close to unity, consistent with single exponential decay. Upon increasing dopant incorporation this value approached 0.5, consistent with a distribution of relaxation times.^56^ It is worth noting, however, that with increasing amounts of Yb^3+^, there is an increase in the confidence intervals for β in Figure 4c, indicating a poorer fit. A comparison of the use of a stretched versus biexponential fit is shown for increasing Yb^3+^ doping concentrations in Figure S7. In the case of a biexponential fit, contribution of a faster relaxing component increases with increasing dopant concentration and starts to dominate the fit above 10%. This supports the evidence of clustering seen in the EDX maps in Figure 3. In the case of a biexponential fit, one *T*_1_ component would be attributed to ^133^Cs nuclei in the bulk lattice, and a shorter component would be attributed to PRE effects on nuclei in the vicinity of an Yb^3+^ aggregate. From these results we can conclude that Yb^3+^ is incorporated into the CsPbCl_3_ perovskite and at higher dopant concentrations Yb^3+^ clustering occurs, which is consistent with the obtained EDX results.

In order to gain more insight as to the source of the PRE effect we compared the spin–lattice relaxation, *T*_1_, of series A at 18.8 and 9.4 T, Figure S6d. We observe a decrease in the spin relaxation times for low ytterbium concentrations at a lower magnetic field. At higher concentrations, 5, 10% and 20% doping, no significant changes in the *T*_1_ are observed with the applied field. The PRE effect arises due to coupling between the unpaired electron of the paramagnetic ion and the neighboring nuclei. Given that unpaired electrons on Yb^3+^ typically have an anisotropic electron g-factor and short electron relaxation times,^57^ the magnitude of the PRE effect likely reflects changes in the electron spin–lattice relaxation time, *T*_1e_. The reduction in the PRE effect at a low concentration and a higher field may reflect an increase in the electron relaxation time through the suppression of hyperfine-mediated relaxation pathways. At higher dopant concentrations, electron dipolar relaxation dominates due to the higher concentration of neighboring electron spins.

#### 3.1.4 ^207^Pb Solid-State Nuclear Magnetic Resonance Spectroscopy

^207^Pb NMR spectra of Yb^3+^ doped CsPbCl_3_ samples mechanosynthesized in agate jars and prepared with excess chloride, series A, are displayed in Figure 5. A single lead site is observed with an isotropic chemical shift of −755 ppm measured at *T* = 240 K. Lead is particularly sensitive to local structural changes as well as temperature, as previous measurements of ^207^Pb in CsPbCl_3_ at 293 K have found chemical shifts closer to −728 ppm.^36^ As the dopant concentration increases we observe an initial narrowing of the central peak in the MAS spectrum rather than broadening as we observe in the ^133^Cs spectrum. In the absence of electron exchange interactions, as the paramagnetic dopant concentration increases, neighboring ^207^Pb nuclei will experience a reduction in *T*_2_ and in *T*_1_ as has been observed for Nd^3+^ doped CsPbCl_3_.^34^ The observed line narrowing is likely a consequence of *T*_2_ filtering due to the use of long echo delays, meaning that fast relaxing components (broader components) of the signal will not be detected in the echo. However, additional measurements showing the effect of variable echo delay are shown in Figure S10, showing no change in line width with reduced echo delay. This is consistent with some lead sites relaxing on a faster time scale than was measurable. The contribution of these faster relaxing components increases as the dopant concentration increases. The remaining narrow signal reflects ^207^Pb sites that are not in close proximity to a paramagnetic ion. In addition, as the amount of dopant is increased, sidebands are observed for this peak, possibly suggestive of an increase in the chemical shift anisotropy or local chemical disorder of these lead sites.

**Figure 5:**
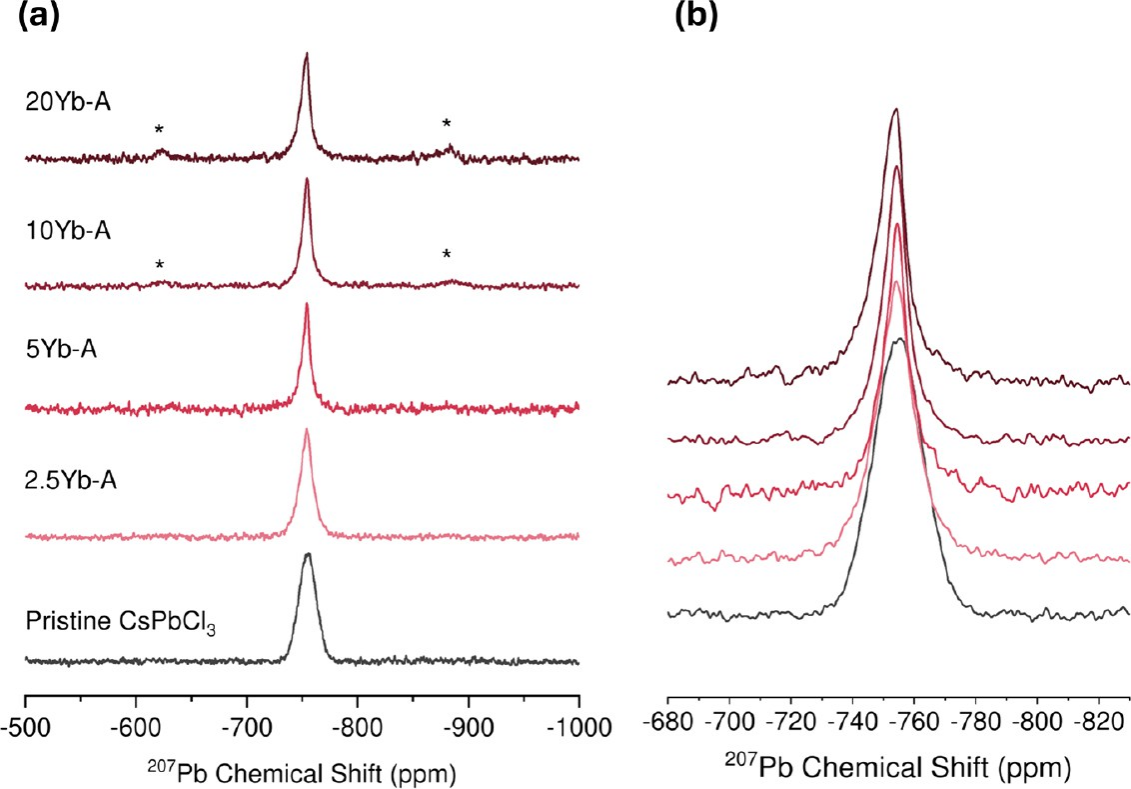
^207^Pb MAS solid-state NMR spectra (18.8 T, 22 kHz, 240 K), (a) at higher amounts of the dopant, side bands appear. (b) One signal can be observed at −755 ppm for all samples.

In order to further investigate the chemical shift anisotropy of the lead site, static ^207^Pb NMR measurements were carried out and fit in order to extract relevant NMR parameters. In the Herzfeld–Berger notation,^58^ the chemical shift anisotropy tensor is described in terms of three parameters, δ_iso_, the isotropic value of the chemical shift, Ω, the span, which describes the width of the signal in a powder spectrum, and κ, the skew, which is a measurement of the asymmetry of the tensor (ranging from −1 to 1). The following equations define these three parameters in terms of the principal components of the chemical shift tensor

2 $$\delta_{\mathrm{iso}}=\frac{\delta_{11}+\delta_{22}+\delta_{33}}{3}$$

3 $$\Omega =\delta_{11}-\delta_{33}$$

4 $$\kappa =\frac{3\left(\delta_{22}-\delta_{\mathrm{iso}}\right)}{\Omega }$$

Figure 6 shows the static NMR spectra for ^207^Pb with a superimposed fit obtained from Dmfit using a single ^207^Pb site model. We observe a nonaxial chemical shift anisotropy (CSA) tensor in the pristine CsPbCl_3_, κ = −0.38, consistent with what we would expect for the lead site in an orthorhombic CsPbCl_3_ structure, due to the tilted Pb–Cl octahedra. The static spectra were fit using dmfit and ssNake and the resulting parameters are shown in Table 2 and Figure S11. As the dopant concentration is increased the static signal broadens significantly. When comparing pristine and 20% doped samples a maximum increase of 40 ppm in the span is obtained.

**Figure 6:**
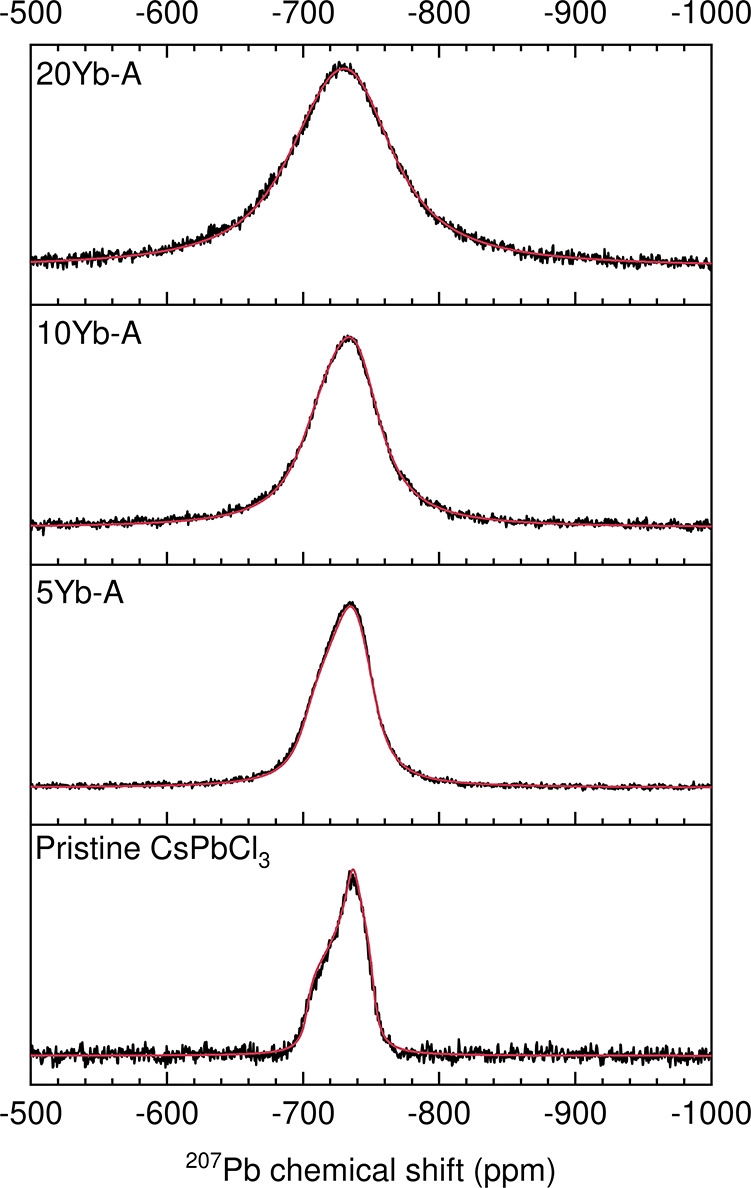
^207^Pb solid-state static NMR spectra (17.6 T, 296 K). Static spectra were collected in order to determine changes in the chemical shift anisotropy as a function of Yb^3+^ doping. Experimental spectra are in black, while the simulated spectra are shown in red.

**Table 2:** Set of NMR Fit Parameters Obtained for ^207^Pb Solid-State Static NMR Spectra (the Values in Parentheses Correspond to 95% Confidence Intervals of the Last Digits)

Sample	δ_iso_ (ppm)	Ω (ppm)	κ (ppm)	Lorentzian broadening (a.u.)
Pristine CsPbCl_3_	–732.84(20)	48.15(87)	–0.380(24)	2873(221)
5Yb-A	–729.42(8)	51.30(17)	–0.361(16)	6868(132)
10Yb-A	–730.10(12)	56.18(100)	–0.278(30)	10,469(263)
20Yb-A	–727.87(17)	81.12(120)	–0.243(28)	12,540(286)

#### 3.1.5 Photoluminescence Quantum Yields

In order to assess the photoluminescence quantum yield (PLQY) of the prepared mechanosynthesized samples, their photoluminescence was measured at room temperature. Figure 7a contains the emission spectra of the samples, with the main band for the Yb^3+^ emission occurring around 985 nm. The shape of the signal for the emission of Yb^3+^ is a consequence of a Stark splitting of the electronic states involved in the transition. Different crystal fields around the lanthanide ion result in a distinct Stark splitting pattern, which is reflected in the distinct peaks and shoulders appearing in the emission signal.^29^ Additionally, electron–phonon coupling leads to broadening of these splittings.^11^ Photoluminescence spectra collected between 900 and 1100 nm shows that all samples studied have a similar splitting pattern and shape at room temperature. Lower temperature PL measurements would be required in order to assess any more subtle changes. On the basis of the intensities of this emission, the PLQYs for each sample were calculated. The resulting values are shown in Figure 7b. We observe a maximum PLQY of 72% with 10 mol % ytterbium. As the concentration of Yb^3+^ in the starting mixture increases, the near infrared (NIR) emission quantum yield increases, except for the higher concentration of 20 mol % where we observe a drop in PLQY. The drop in the PLQY is explained by the aggregation of ytterbium observed in the EDX maps: concentration quenching may occur via cross relaxation, a process in which neighboring emitters transfer energy to each other nonradiatively, contributing to the decrease in the observed PLQY.^59,60^ As shown in Figure 7b we observe a gradual decrease in *T*_1_ with increasing PLQY until we reach a relaxation time below 10 s at 20% doping at which point the PLQY is seen to decrease significantly. We note that the contribution of the fast relaxing component in biexponential *T*_1_ fits of the Yb^3+^ doped samples becomes dominant in 10% and 20% doped samples, Figure S7. This biexponential behavior is consistent with the observed Yb aggregation in the EDX measurements as well as the decrease in the observed PLQY.

**Figure 7:**
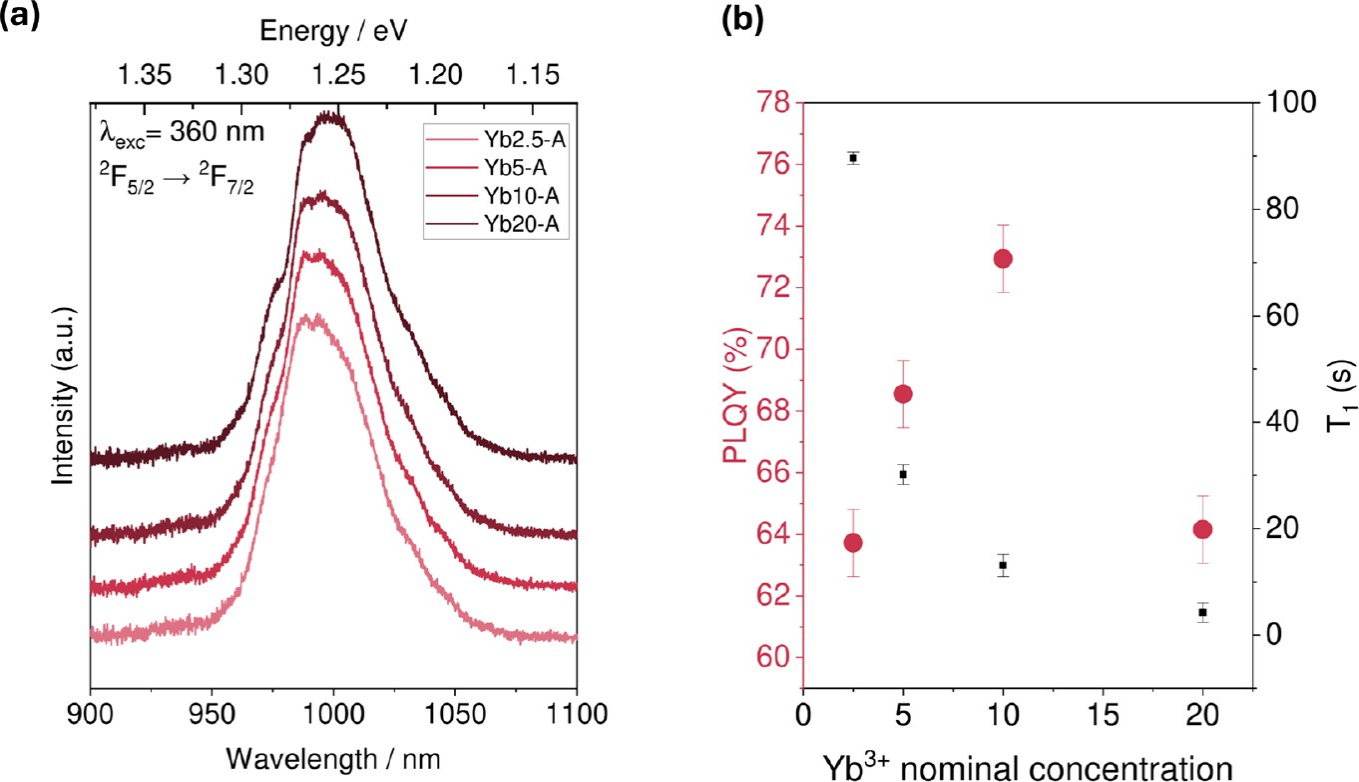
(a) Photoluminescence spectra of the 2.5, 5, 10, and 20% doped samples prepared using mechanosynthesis with excess chloride ion (series A). The emission in the NIR region, due to the ^2^F_5/2_ → ^2^F_7/2_ electronic transition of Yb^3+^, the emissive feature is centered around 985 nm, and with a zero-phonon line at 976 nm.^60^ (b) Photoluminescence quantum yields (red) for the NIR emission of the ytterbium-doped perovskites compared to *T*_1_ measurements at identical concentrations (black) (series A) (The error bars indicate the standard deviation in PLQY obtained from a repeated measurement on an identical sample).

### 3.2 Effects of Chloride Concentration

One of the proposed defects relevant for PLQY in CsPbCl_3_ is thought to form a dimer complex that displaces three Pb sites, Yb^3+^-V_Pb_-Yb^3+^. In the case of series A, we investigated the role of excess chloride to facilitate Yb–Cl incorporation using an excess of chloride greater than 80 μmol in all samples. In the series described in this section, series B, we prepare a series of Yb^3+^ doped samples prepared with depleted PbCl_2_, with a minimum depletion of 40 μmol in order to investigate if lead depletion assists in dimer formation and subsequently improved PLQY. To this end, we mechanosynthesized Yb^3+^ doped samples in agate jars, with the same increase in Yb^3+^ concentrations as series A but with depleted PbCl_2_ in order to reach a stoichiometric ratio of chloride, as well as a depletion of lead. Lastly, given the reported increase in PLQY with the use of excess metal halides in the preparation of nanocrystals,^61^ we also prepared a 5 mol % Yb^3+^ series with depleted lead (stoichiometric chloride—C1), stoichiometric lead (excess chloride—C2) and excess lead (greater excess chloride—C3) which we label as series C. Only slight differences in stoichiometric ratios were explored in order to avoid significantly perturbing the formation of the nominal CsPbCl_3_ perovskite structure. These latter three samples were prepared using stainless steel jars and balls rather than agate materials. Further details regarding reagent ratios and choice of depletion values are included in the SI.

#### 3.2.1 X-ray Diffraction

The powder XRD results for the perovskite samples of series B as well as series C are shown in Figure 8. In all samples one major phase is observed in the pXRD which is in good agreement with the reference pattern of the orthorhombic cesium lead chloride perovskite phase. However, a small contribution from a secondary phase was observed in all samples from series B as well as in sample C1. Phase indexing revealed that these peaks are consistent with the Cs_4_PbCl_6_ reference diffraction pattern, corresponding to a nonperovskite phase. The formation of Cs_4_PbCl_6_ has been reported to occur in cesium rich conditions with an excess of a cesium precursor being used to produce a cesium rich phase.^62^ The appearance of this phase is likely due to the depleted lead composition of series B and sample C1 leading to cesium rich regions during the solid-state synthesis. Notably this phase is absent for the series C2 and C3 samples. We do, however, observe the appearance of weak peaks associated with PbCl_2_ in the C3 sample which includes an excess of both the lead and chloride ion, Figure 8b. Notably, at higher doping concentrations in series B, at 10% and 20% doping the diffraction maxima associated with the Cs_4_PbCl_6_ shift to lower angles (consistent with lattice expansion) and some of the characteristic peaks associated with the Cs_4_PbCl_6_ reference diffractogram disappear. We propose that these peaks are associated with an Yb rich low dimensional perovskite Cs_4_PbCl_6_-like phase that forms under cesium rich, lead poor conditions and high Yb doping concentrations.

**Figure 8:**
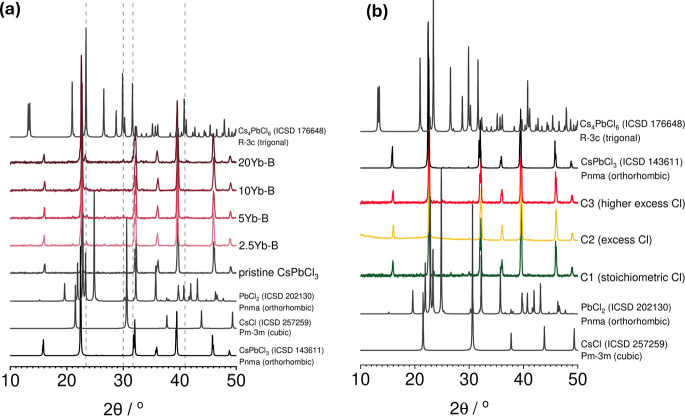
(a) X-ray diffraction powder patterns of series B (stoichiometric chloride). From bottom to top, the patterns correspond to the simulated reference powder pattern for orthorhombic CsPbCl_3_ (in black),^49^ the pristine mechanosynthesized CsPbCl_3_ sample and the doped samples (in shades of red), and the simulated reference powder pattern for Cs_4_PbCl_6_ (nonperovskite phase, in gray).^63^ The vertical gray lines highlight the peaks at 23.4°, 30.0°, 31.7°, and 40.9°, which match the latter reference as a minor secondary phase synthesized during the process. (b) XRD powder patterns for the perovskite samples mechanochemically synthesized with different stoichiometric ratios of PbCl_2_ and CsCl using stainless steel jars.

#### 3.2.2 ^133^Cs Solid-State Nuclear Magnetic Resonance Spectroscopy

All series B and series C samples exhibit a peak at 83.8 ppm consistent with the orthorhombic CsPbCl_3_ phase, with gradual broadening observed at increasing doping concentrations as observed in series A. Samples C2 and C3 show a pure phase spectrum with only a ^133^Cs site in a CsPbCl_3_ phase observed. In sample C1 (lead depleted) as well as series B 2.5% and 5% Yb doped samples a peak at 179 ppm is observed in the ^133^Cs NMR spectra, Figure 9, consistent with the known nonperovskite phase Cs_4_PbCl_6_ chemical shift.^33^ We also observe a small broadened peak appearing at 230 ppm as well as a broadened peak at 52 ppm in the 5% doped sample, these peaks are not observed for samples prepared with stainless steel grinding (C1), where only the Cs_4_PbCl_6_ shift is observed. These peaks only appear at higher Yb doping concentrations and increase in intensity as the doping increases to 20%. This increase is accompanied by a disappearance of the peak at 179 ppm. *T*_1_ relaxation measurements were taken of these additional peaks and it was found that as compared to the pure nonperovskite phase Cs_4_PbCl_6_ with a *T*_1_ of 80 s, these ^133^Cs sites exhibit much shorter relaxation times of 1 s for the peak at 52 ppm and 2 s for the peak at 230 ppm, as seen in Figure S13, consistent with paramagnetic doping. We note that these peaks are absent in the series A sample which also does not contain any lower dimensional perovskite components in both the NMR and PXRD at higher doping concentrations. We propose that these peaks are associated with ^133^Cs sites in an Yb doped nonperovskite Cs_4_PbCl_6_-like phase. Correlation measurements using a standard NOE sequence^64^ that were performed indicate no exchange of magnetization between the sites, consistent with ^133^Cs sites in distinct phases, shown in Figure S14.

**Figure 9:**
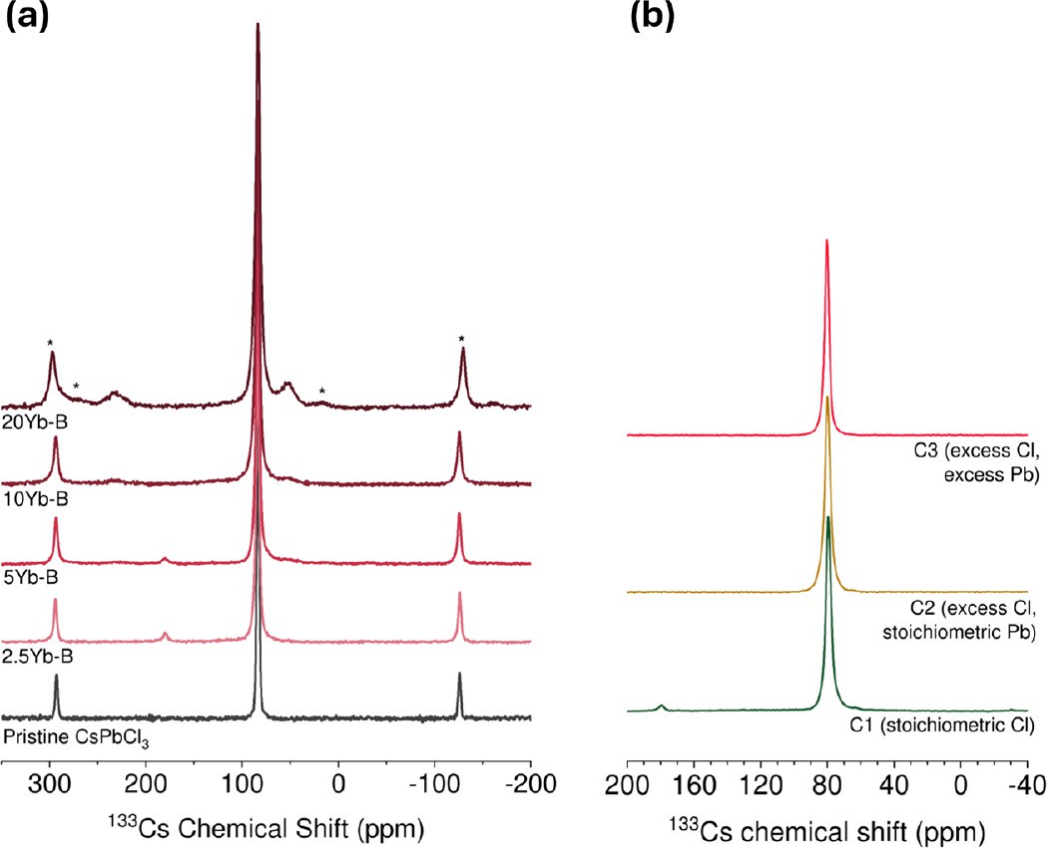
^133^Cs NMR spectra (18.8 T, 240 K, 22 kHz) of (a) CsPbCl_3_ perovskite samples synthesized with depleted lead (series B). and (b) CsPbCl_3_ perovskite samples synthesized with depleted PbCl_2_ (as in series B, generating stoichiometric amounts of Cl), 1:1 stoichiometric PbCl_2_ (as in series A, with excess amounts of Cl), and excess PbCl_2_ (generating a higher excess of Cl) (using stainless steel jar).

As in series A, we likewise measured the spin–lattice relaxation times of the observed ^133^Cs sites and found that the *T*_1_ values of the ^133^Cs site in CsPbCl_3_ were consistently longer for series B samples at higher doping concentrations than the case for series A, Figure 10. This indicates that more of the Yb^3+^ is incorporated into the CsPbCl_3_ perovskite lattice in series A samples rather than series B. This is consistent with Yb^3+^ associating with lower dimensional phases in series B samples. The same trend is observed in the β parameter, where series A exhibits lower β values approaching 0.5 as compared to series B.

**Figure 10:**
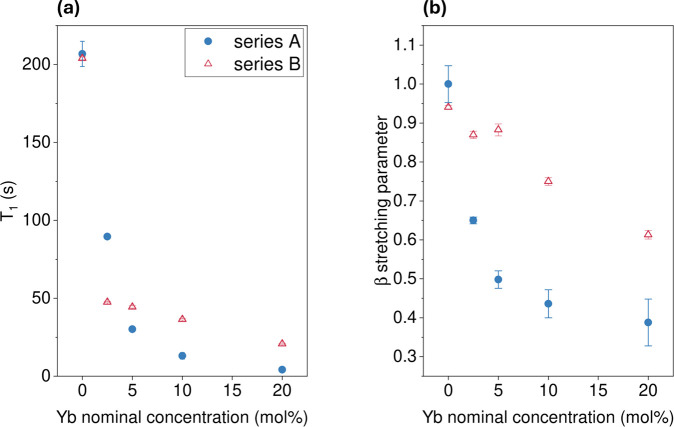
*T*_1_ relaxation time-constants (a) and β stretching parameter (b) for ^133^Cs nuclei in the perovskite samples synthesized with 1:1 stoichiometric ratio of the starting materials (series B) are shown in red. For comparison, the corresponding values for series A are shown in blue.

#### 3.2.3 Photoluminescence Quantum Yields

The obtained PLQY results are shown in Figure 11b. Qualitatively, all PLQY spectra exhibit a similar profile as described in the previous section, indicative of a similar electronic environment around the lanthanide ion.

**Figure 11:**
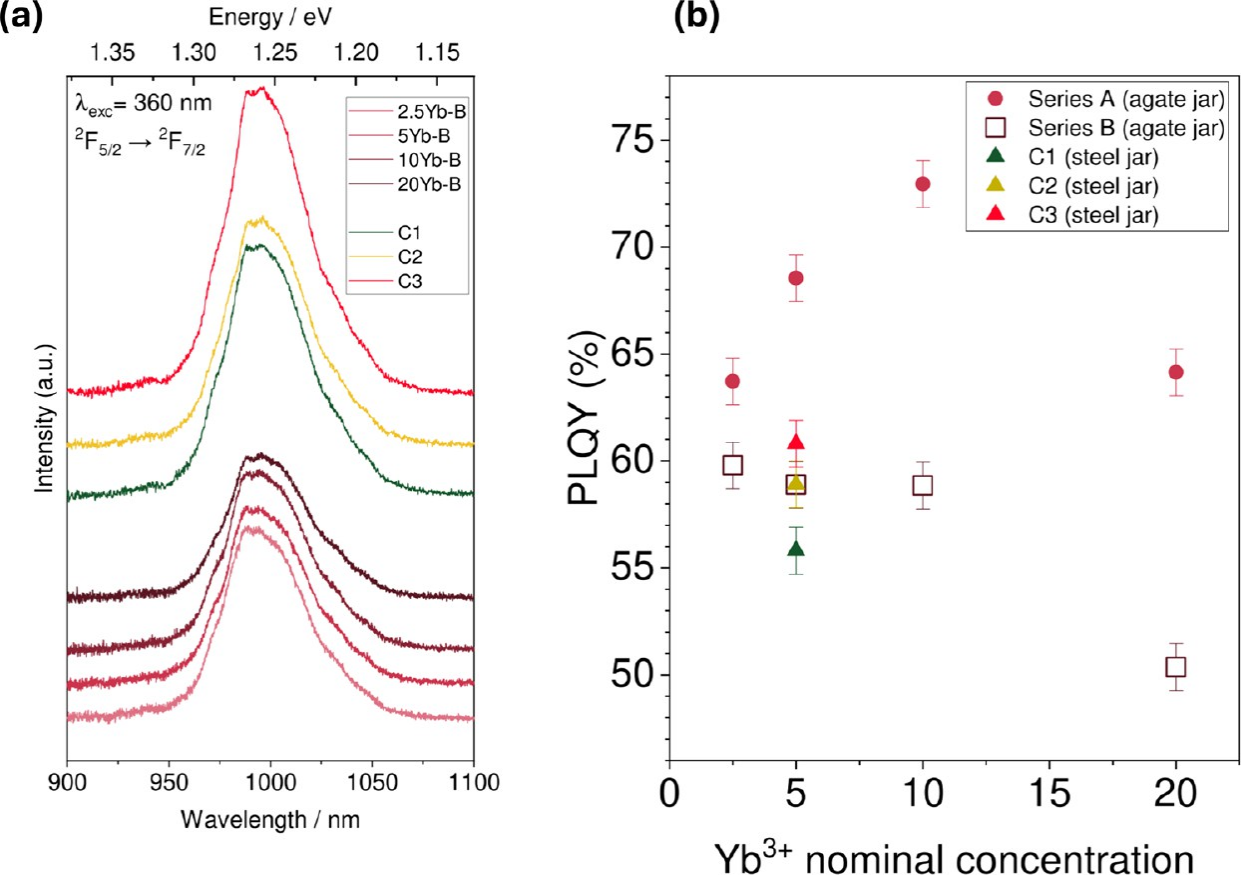
(a) Four lower photoluminescence spectra correspond to Yb-doped samples from series B prepared using agate jars; the three upper profiles are from samples with increasing chloride content, synthesized using stainless steel jars. (b) Comparative plot of the PLQYs for series A and B, and series C samples synthesized using steel jars. The error bars indicate the standard deviation in PLQY obtained from a repeated measurement on an identical sample.

The NIR quantum yields for series B prepared in agate jars were between 55 and 59%, while for the case of varied chloride concentration prepared in stainless steel jars the PLQYs varied from 55 to 60%, with the highest value obtained for the sample with the greatest excess of PbCl_2_. In Figure 11b we also include the results from series A prepared in agate jars where excess chloride concentrations were used across the Yb^3+^ doping series. We note that the PLQYs of series A are consistently larger than those of series B as well as larger than those of the samples prepared using stainless steel jars. This suggests that using agate jars for mechanochemical preparation along with using excess chloride will likely provide Yb^3+^ doped CsPbCl_3_ powders with larger PLQYs. We originally anticipated that the use of lead depletion would facilitate the formation of lead vacancies and therefore result in greater formation of the relevant Yb^3+^-V_Pb^2+^_-Yb^3+^ species upon doping. However, as seen from the pXRD and NMR results, preparing samples with stoichiometric chloride and depleted lead, rather than improving the PLQY, leads to the formation of Cs_4_PbCl_6_ that has a detrimental effect on the PLQY, partly due to the reduced incorporation of the doped Yb^3+^ into the perovskite phase CsPbCl_3_.

### 3.3 Effects of Increased Grinding Times

In addition to promoting the reaction, the mechanical shock between the particles within the material once it is synthesized promotes the introduction of additional defects—both point defects and higher dimension defects, such as grain boundaries and dislocations. In addition, grinding has the effect of reducing particle sizes which has been suggested to affect the QC mechanism in this class of systems. The effect of grinding involves a complex interplay of defect formation and annealing and has been shown in some cases to improve the properties of a material.^41^ To evaluate how increasing grinding times may affect the formation of defects, the size of the particles and the PLQYs, samples at a fixed concentration of 5 mol % of Yb^3+^ were prepared, followed by increased grinding times between 30 min and 6 h. The grinding was paused for at least 5 min after each hour of grinding. A premix of the starting materials was added to a single jar and a small amount was extracted under N_2_ atmosphere at increasing grinding times (series G2). These samples were compared to a set of materials prepared separately for each grinding time (series G1).

#### 3.3.1 Scanning Electron Microscopy

In order to assess the effect of grinding on the samples at increased ball-milling times, SEM images were acquired for each of the grinding times prepared in series G2. These are shown in Figure 12. It can be noted from these images that, as the grinding time increases, the morphology of the samples changes, going from a more regularly shaped crystal habit to more uneven, distorted grains. This is a direct result of ball-milling, which crushes and breaks apart the formed crystals through mechanical shock, leaving them with irregular shapes.

**Figure 12:**
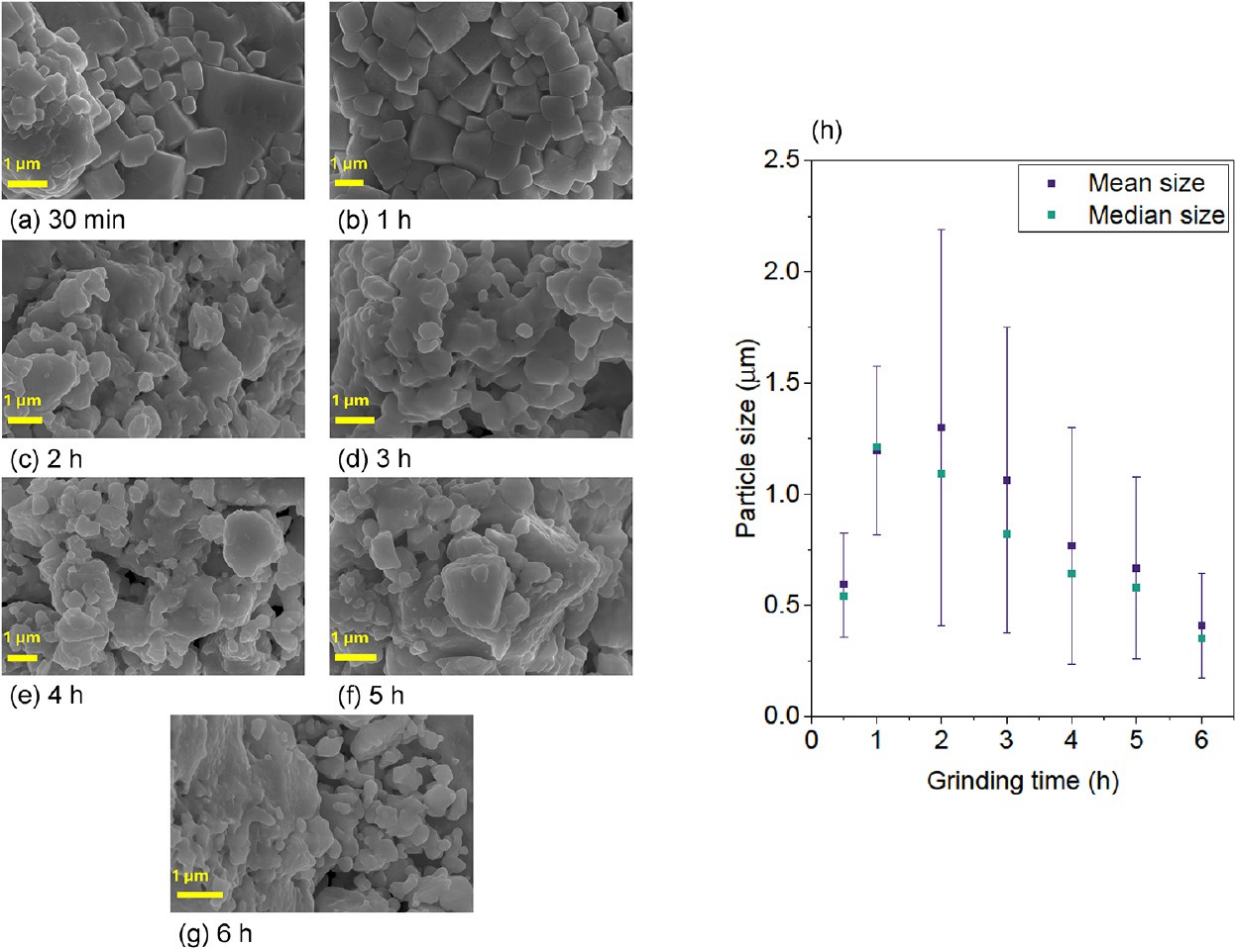
SEM images from CsPbCl_3_:5Yb^3+^ (series G2) showing details of the morphology of the samples extracted at increasing grinding times (a–g); variation of particle sizes as a function of grinding time (h).

Furthermore, there is a size reduction effect with increasing ball-milling time, as is expected from this method. This can be observed in Figure 12h. There is an initial increase in the particle mean size in the first two hours of grinding, followed by size reduction with further milling. The initial size increase is suggestive of the mechanical energy being utilized in the synthesis of the product, and once the perovskite phase is fully formed, the energy provided by subsequent ball-milling is then used to reduce the size of the formed product. As it is later shown by XRD and NMR analysis, the perovskite is obtained as a single phase starting at 2 h of grinding, which corresponds to the largest particle mean size observed.

The large standard deviation of the values (visually represented by the error bars in the plot), indicates the large size distribution of the particles in the sample, which can be observed in the micrographs in Figure 12. This is, again, a result from the mechanical shock imparted by the dry ball-milling method, which results in a large distribution of particle sizes. Following 6 h of grinding time we obtained a median size of 352 nm.

#### 3.3.2 X-ray Diffraction

Powder X-ray diffraction results of series G1 and G2 for the distinct grinding times are shown in Figure S15. In the case of series G1, all samples indicate CsPbCl_3_ as the dominant phase with some trace amounts of the nonperovskite phase Cs_4_PbCl_6_ present in the case of the 30 min, 4 and 5 h which relates to small stoichiometric fluctuations that arise in the weighing of starting materials. Notably with increased grinding time the PXRD peaks remained of a similar width indicating that the crystalline structure is robust against mechanical deformation. In the case of series G2, all samples likewise indicate CsPbCl_3_ as the dominant phase. Preparation of series G2 involved the premixing of a larger amount of reagents which may have led to inhomogeneous mixing effects as seen by the production of additional phases (as seen in Figures 13 and S15) at shorter milling times, and an incomplete reaction. At longer milling times the contribution of the nonperovskite component is significantly reduced and the CsPbCl_3_ component dominates.

**Figure 13:**
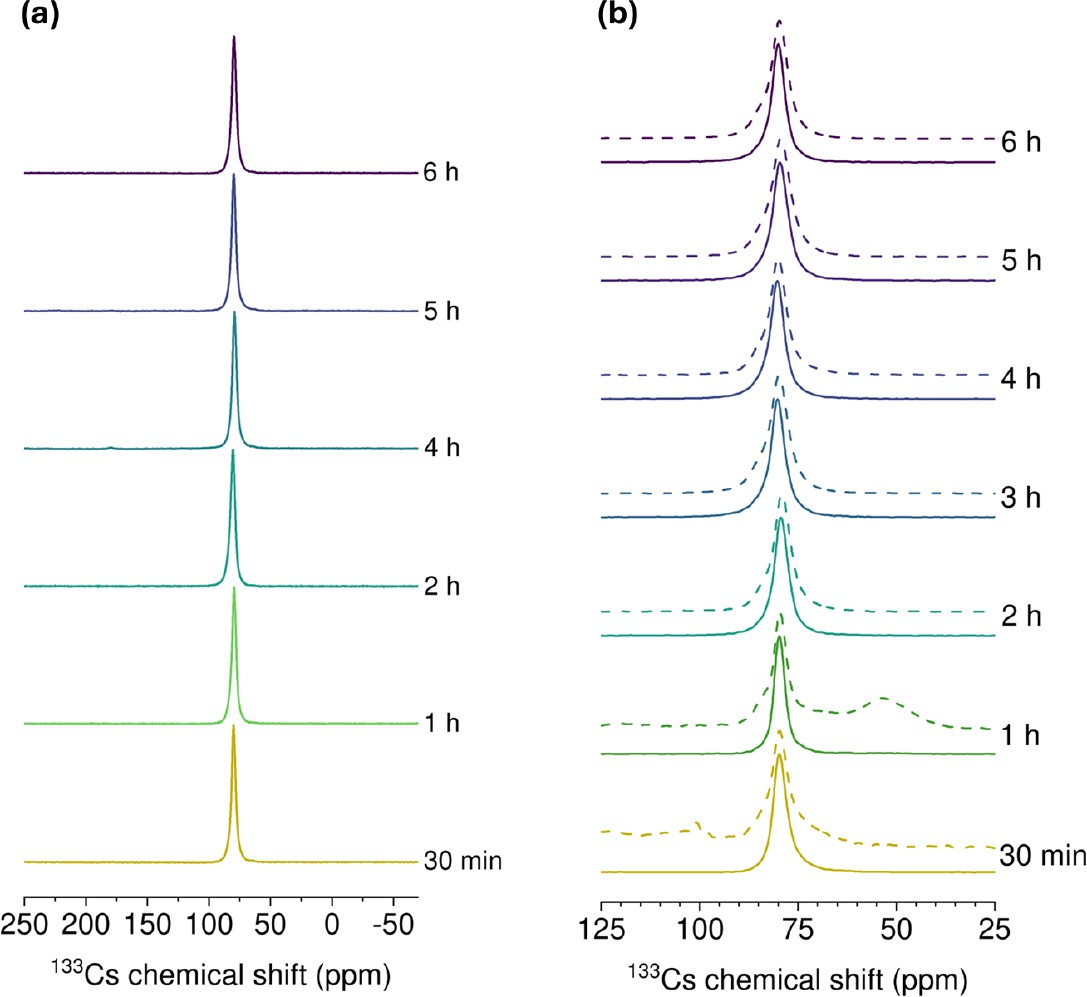
^133^Cs solid-state static NMR spectra for samples with different grinding times, (a) series G1, (b) series G2. Solid lines correspond to quantitative spectra, while the dashed lines are spectra taken with short recycle delays (all samples measured at 18.8 T 260 K, 22 kHz).

#### 3.3.3 ^133^Cs Solid-State Nuclear Magnetic Resonance Spectroscopy

The ^133^Cs NMR spectra in Figure 13 show that, for series G2 at 30 min and 1 h of ball-milling, the samples are not composed of only one single phase, in contrast to the individually synthesized samples where only the ^133^Cs site associated with the orthorhombic phase is observed. (series G1, Figure 13a). This is consistent with our pXRD results. To better visualize the different features in the spectra, shorter recycle delays (1 s) were used during the acquisition of the spectra (dashed lines in Figure 13). The series G2 samples with synthesis times of 2 h and longer showed one feature consistent with an orthorhombic phase of CsPbCl_3_ at 80.5 ppm. As seen from our pXRD results, a few samples contain a trace amount of the nonperovskite phase Cs_4_PbCl_6_. This feature appears at 178 ppm in the ^133^Cs spectra and corresponds to the cesium-rich phase Cs_4_PbCl_6_. However, additional ^133^Cs sites can be identified in those samples, using the fast recycling delay approach: a broad feature at 218 ppm, at 115 and 53 ppm. The disappearance of all features with longer milling times indicates that additional cesium containing phases were converted into CsPbCl_3_, this is also supported by our XRD results above.

As we compare the ^133^Cs lineshapes from low to high grinding times no change in line width is observed. Furthermore, a comparison of 2 and 6 h grinding-time NMR spectra taken at a lower spin rate of 3 kHz (Figure S17) showed there was little to no change to the chemical shift anisotropy of the cesium site as grinding time is increased. If we next compare the spin–lattice relaxation times, *T*_1_ at 18.8 T, of the ^133^Cs resonance at 80 ppm for series G2 (Figure 14) we observe a decreasing trend in spin–lattice relaxation times at longer grinding times. Likewise we observe a reduction in the PLQYs at higher grinding times, reaching a maximum PLQY around 1 to 2 h of grinding.

**Figure 14:**
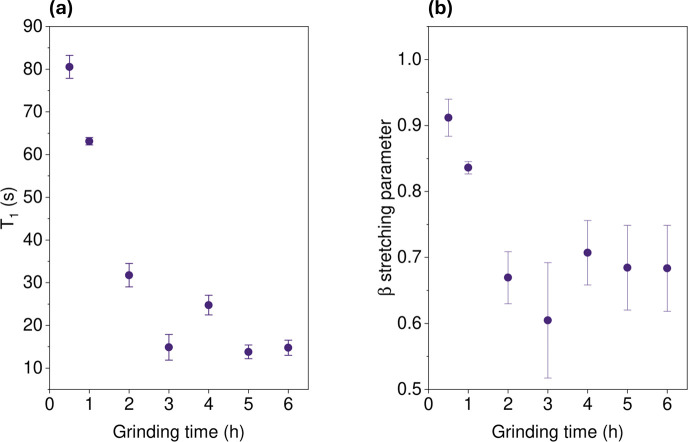
^133^Cs *T*_1_ relaxation for series G2 of CsPbCl_3_:5Yb^3+^ at increasing grinding times (a) and its respective β stretching parameter from saturation recovery fits (b).

#### 3.3.4 Photoluminescence Quantum Yields

Figure 15 shows the PLQYs for the NIR emission at increasing grinding times. The corresponding PL spectra are found in the SI, in Figure S19. An initial increase in PLQY is observed, reaching a maximum value of 78.3% with 2 h of grinding time in the case of series G2. Further grinding beyond 2 h only served to decrease the observed PLQY. We note that the highest PLQY value in series G1 was observed after 1 h of grinding and the ^133^Cs site in CsPbCl_3_ was measured to have a *T*_1_ of 22 s while that of series G2 the highest PLQY value was observed after 2 h of grinding time and had a *T*_1_ of 31 s. The relationship between the magnetic resonance relaxation behavior and the PLQY on the samples from series G2 can be visualized in Figure 17a: additional defect sites (such as the aforementioned paramagnetic point defects, but also higher dimensional defects) may have contributed to the observed decreasing trend in *T*_1_ relaxation with increased grinding time, as well as the possible introduction of nonradiative decay pathways, thus reducing the NIR emission. The observation of a maximum PLQY after a particular grinding time is likely related to the reduction of secondary phases in the samples.

**Figure 15:**
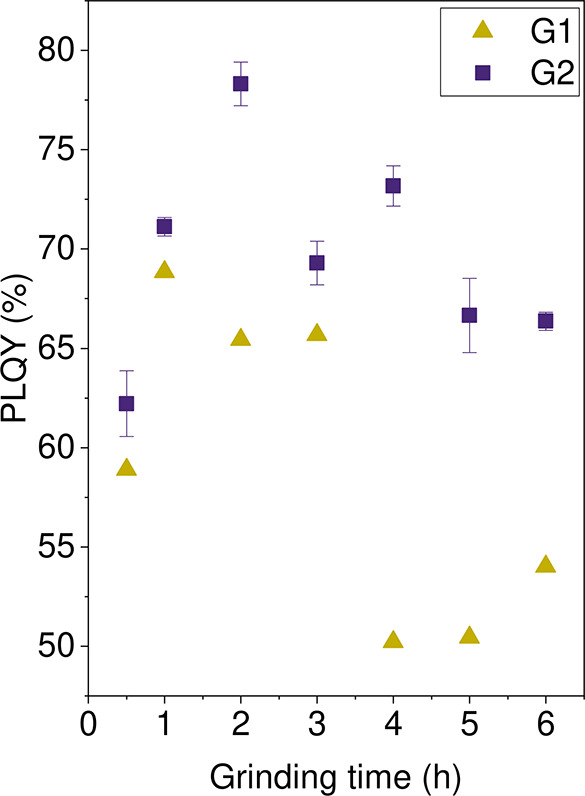
PLQY for the Yb-doped CsPbCl_3_ spectra (series G1, yellow triangles). A maximum of 78.32% was obtained for the sample with 2 h of synthesis time (series G2, purple squares).

## 4 Discussion

The incorporation of Yb^3+^ cations into the perovskite crystal lattice comprises a case of heterovalent doping, which inherently causes intrinsic defects to form in the structure. These appear in the form of vacancies at some Pb^2+^ sites, due to similarities in ionic radii. It has been reported that the limited lattice shrinking upon incorporation of the smaller Yb^3+^ relative to Pb^2+^ may arise due to combined charge and strain compensation between lead vacancies (V_Pb_^2–^) and ytterbium cations (Yb^3+^) replacing lead in the lattice (V_Pb_^2–^ + Yb^3+^).^25^ However, across all samples studied, we note that the doping process does not promote dramatic changes in the bulk crystal lattice. Techniques that probe long-range order, such as X-ray diffraction, are not sensitive to local structural changes induced by defects in defect tolerant materials.

As we have seen from our results, the ^133^Cs chemical shift is sensitive to changes in the local magnetic environment. We observe broadening and a skewed-Gaussian line shape at higher doping concentrations in both series A and B, as seen for series A in Figure 4b as well as in Figure S6. In addition, we observe no increase in broadening with increased grinding times in series G at a fixed doping concentration. The observed broadening and shape may arise due to several factors, which we consider in the following discussion. If we consider broadening due to changes in the quadrupolar splitting parameter, we can note that ^133^Cs has one of the smallest electric quadrupole moments among all quadrupolar nuclei (−0.343 fm^2^).^65^ At moderately high fields, where the Zeeman interaction is significantly larger than the quadrupole interaction it can be treated as a pseudospin-1/2 nucleus; therefore, broadening due to quadrupolar couplings is unlikely in this case. If we consider the effect of the paramagnetic dopants, a higher concentration of paramagnetic dopants may lead to a shorter *T*_2_* which can lead to inhomogeneous broadening of the line shape. If we compare the *T*_2_* values of a 20% doped versus that of a 5% doped we see a drop in the *T*_2_* from 806 ± 5 μs to 502 ± 4 as shown in Figure S5. We also consider the possibility of multiple sites leading to the observed skewed-Gaussian shape, for example as due to pseudocontact shifts (PCS) as has been seen for other lanthanide doped perovskites.^34^ Given that the g-tensors of Yb^3+^ ions are typically anisotropic, the asymmetry in the line shape may arise due to unresolved PCS.^66^ If this is the case we would expect to observe a distortion in the ^133^Cs line shape when we compare short and long recycle delay measurements of the 20% sample. This distortion in the line shape can be seen in Figure 16.

**Figure 16:**
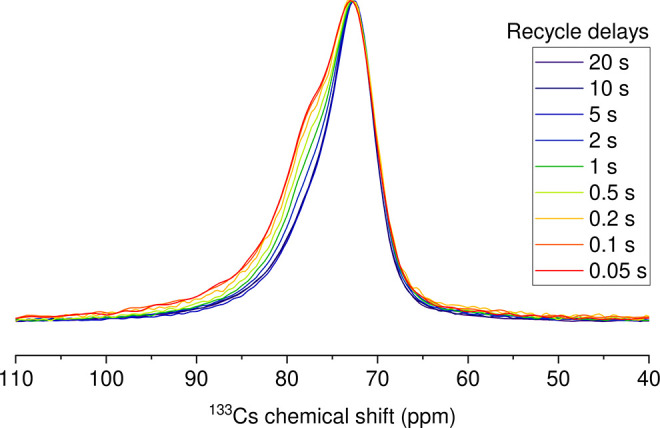
^133^Cs NMR for the 20Yb:CsPbCl_3_ sample from series A measured at different recycle delays. The asymmetric broadening of the line shape at fast recycling conditions indicates the inclusion of sites with faster relaxation to the distribution of chemical shifts shown, due to pseudocontact shifts. All spectra were measured at 9.4 T, with 22 kHz of spin rate (measurements at 18.8 T are shown in Figure S8).

PCS arises from through-space coupling between the electronic and nuclear magnetic dipoles and scales as *r* ^–3^ with respect to the electron–nuclear distance.^34,66^ As the amount of doping increases, the average distance between Yb^3+^ ions and ^133^Cs atoms is reduced and the extent of the skewed-Gaussian line shape increases. And as shown in Figure 16 additional resonances can be seen more clearly when measuring ^133^Cs NMR under fast recycle delays. The magnitude and sign of the observed PCS depends on the anisotropy and asymmetry of the paramagnetic ion’s magnetic susceptibility tensor, χ, as well as the ion’s proximity and orientation relative to the ^133^Cs nucleus. The sign of the PCS can be determined from how the chemical shift of the observe nucleus changes in the presence of the paramagnetic dopant, for example, a positive PCS leads to a shift to higher chemical shifts while a negative PCS leads to a shift to lower chemical shifts.^66^ The observed chemical shift due to PCS can be described as^67^

5 $$\Delta \delta^{\mathrm{PCS}}=\frac{1}{12\pi r^{3}}\left[\Delta \chi_{\mathrm{ax}}\left(3\cos^{2}\theta -1\right)+\frac{3}{2}\Delta \chi_{\mathrm{rh}}\sin^{2}\theta \cos2\phi \right]$$

Here, Δχ_ax_ is the axial component, Δχ_rh_ is the equatorial (rhombic) component of the magnetic susceptibility tensor of the paramagnetic ion and (*r*, θ, ϕ) represent the relative position of the nucleus in the principal-axis frame of the magnetic susceptibility tensor χ.

In the case of an Yb^3+^ paramagnetic ion, if Δχ_ax_ and Δχ_rh_ are both negative, as has been reported for other Yb^3+^ species,^67^ a positive PCS is consistent with a nucleus being in the equatorial plane of the susceptibility tensor. It is important to note that the paramagnetic species that contributes to PCS in this case could also be a more complex ytterbium defect that may involve vacancies or an additional ytterbium ion.^25^ Recent EPR studies on 5% Yb^3+^ doped CsPbCl_3_ indicate a spin 1/2 species with an orthorhombic g-tensor and in close proximity to a ^133^Cs nucleus which the authors attribute to a monomeric Yb^3+^ species. The authors also observe additional EPR lines that the authors suggest relate to a dimeric Yb^3+^ species.^68^ The shifts and skewed-Gaussian line shape observed in highly doped ^133^Cs samples (Figure 16) are consistent with a positive PCS.

Additional contributions to the broadening could arise from anisotropic bulk magnetic susceptibility (ABMS) effects, as has been observed for erbium doping at low concentrations, a paramagnetic ion with significantly larger anisotropic magnetic susceptibility tensor, χ, than ytterbium.^34,69^ ABMS effects arise due to local magnetic field distributions across a polycrystalline sample.^70,71^ As described by Schwerk et al.,^71^ differences in packing of the crystallites leads to observed global broadening effects due to differences in the intercrystallite dipolar fields. For example, broadening could occur due to differences in intercrystallite dipolar fields across the sample as well as from contributions of different paramagnetic compositions in the crystallites within the sample, particularly at higher doping conditions. For example, EDX results indicate clustering in samples above 5% doping and as seen from Figures 4 and S6d in the SI, the line broadening increases significantly from 5% to 10% to 20%. However, temperature dependent measurements on 5% samples indicated a flat response in terms of line width (Figure S4). Given that magnetic susceptibility would have a marked temperature dependence as well as a uniform broadening across the spectral line shape, ABMS is not the dominant contributor to the observed broadening.

This broadening with increasing dopant concentration could instead be suggestive of an increase in the distribution of cesium environments upon doping. Previous studies on ytterbium doped cesium lead halides suggest that doping introduces lattice distortion at cesium sites along with a distribution of Cs–Cl bond distances.^27^ In this case, one could expect Gaussian broadening about a particular chemical shift consistent with positional disorder of the Cs atom in addition to a change in the shielding anisotropy.^72^ In summary it is likely that PCS, shielding anisotropy and positional disorder of the Cs atom (which would also lead to changes in *T*_2_*) are the dominant contributors to the ^133^Cs line shape in pure phase samples studied here.

Next we turn our attention to the observed ^207^Pb NMR chemical shifts and spin relaxation behavior for series A and B. For both series we observed a slight increase in the chemical shift (greater deshielding) with increasing ytterbium concentrations. If we compare isotropic shifts extracted for series A from static ^207^Pb spectra (−732 to −727 ppm) and those under spinning conditions, 22 kHz, (−755 to −753 ppm) both show an increase in the chemical shift with increasing Yb^3+^ concentration. This deshielding effect could be consistent with a change in Pb–Cl bonding behavior. Previous X-ray absorption spectroscopy measurements have suggested that doping of Yb^3+^ leads to the lengthening of Pb–Cl bonds.^27^ We note that given that ^207^Pb chemical shifts are particularly sensitive to temperature, the difference in isotropic chemical shifts between the static and spinning cases likely arise due to the temperature differences between the measurements. Fits of static ^207^Pb NMR spectra indicate an increase in the span of 40 ppm at higher doping concentrations as well as a slight decrease in the skew from −0.38 to −0.24. At higher doping concentrations under magic angle spinning at 22 kHz we also observe the appearance of sidebands, consistent with an increase in the CSA of the lead site. However, we observe narrowing of the central ^207^Pb resonance line width which we attribute to the disappearance of signals in close proximity to Yb^3+^ ions due to fast spin–spin relaxation. Unlike in the case of ^133^Cs NMR measurements, PCS contributions were not observed in the ^207^Pb spectrum, potentially due to very short spin-relaxation times of these species. In addition, the spin–lattice relaxation times slightly decrease with increasing Yb concentrations (Figure S9) but the decrease is within the error of *T*_1_ fits. This indicates that we are likely only detecting ^207^Pb sites that are not significantly perturbed by the Yb^3+^ doping aside from slight changes in the shielding and local Pb–Cl bond lengths. Therefore *T*_1_ measurements of the ^207^Pb site were not as effective as ^133^Cs *T*_1_ measurements for probing Yb^3+^ incorporation.

As part of this study, we sought to identify the relationship between the spin–lattice relaxation time *T*_1_, the PLQY and the synthesis approach of Yb doped CsPbCl_3_. In general across all series, for a given ytterbium concentration we observed lower PLQYs in samples that were not composed of a pure phase. In these cases, it was difficult to perform a direct comparison between samples as they had slightly different phase compositions. When comparing PLQY and *T*_1_ we only focus on pure phase samples as shown in Figure 17.

**Figure 17:**
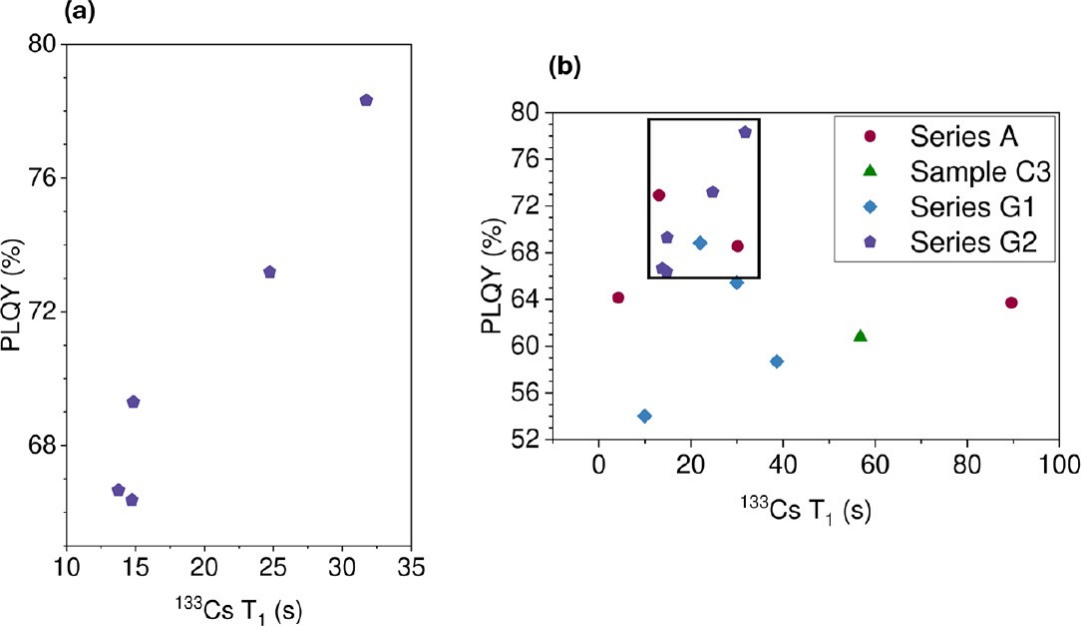
Relationship between PLQY and ^133^Cs *T*_1_ relaxation for the samples in series G2 (a). For comparison, (b) shows the PLQY/*T*_1_ relationship for all samples that showed a pure phase.

*T*_1_ is affected by the concentration and distribution of paramagnetic centers in the lattice via PRE, and the paramagnetic centers are responsible for the NIR emission in the matrix, so, it would be natural to expect a relationship between these two properties. As seen in Figure 17b, although a direct relationship could not be established, higher PLQYs were achieved in series A and series G samples. Both these sets of samples were prepared using an excess of chlorine in the synthesis. Among these samples, the highest PLQYs were observed for series A and G samples with ^133^Cs relaxation times between 13 and 35 s at 18.8 T. A shorter *T*_1_ would arise at high concentrations where concentration quenching would limit the PLQY. For the case of an upper limit on *T*_1_, the drop in PLQY at longer *T*_1_ may be related to the lower probability of Yb–V_Pb_-Yb pairs at reduced Yb^3+^ dopant incorporation. However, it must be kept in mind that, as has been shown throughout this work, other factors play a role in the overall properties of the synthesized materials, such as precipitation at higher dopant concentrations (leading to PL quenching) and generation of secondary phases (that might include the dopants, thus reducing PLQY). Additionally, to verify if the particle size affects the spin–lattice relaxation or optical behavior of the material, in the SI we show a statistical correlation between these three properties (Figure S20). The higher coefficient of determination (*R* ^2^) implies that ^133^Cs *T*_1_ and PLQY are indeed more strongly correlated than PLQY and particle size. Lastly, we note that in our series A study, the highest PLQY was achieved at 10% doping, consistent with what has been observed in other Yb^3+^ doped CsPbCl_3_ samples.

## 5 Conclusions

In this study, we synthesized ytterbium-doped cesium lead chloride using dry ball-milling, with the goal of investigating the incorporation of Yb^3+^ ions into the perovskite lattice and how varying the synthesis conditions affects the structure of the defect site and the photoluminescence activity of the material. A maximum PLQY of 78% was obtained using an excess of chloride and when the grinding time is set to 2 h. Furthermore, larger PLQYs were generally achieved by using a 1:1 stoichiometric ratio of the starting materials CsCl and PbCl_2_ along with 10 mol % YbCl_3_ doping, which lead to samples with an excess of Cl^–^ ions. This stoichiometric ratio was also the optimal ratio to increase the purity of the synthesized phase. The magnetic resonance properties of the nuclei ^133^Cs and ^207^Pb were additionally analyzed to investigate changes in the perovskite structure. As the concentration of the dopant ion was increased, paramagnetic pseudocontact shifts causes broadening and skew on the line shape of ^133^Cs NMR spectra. Paramagnetic relaxation enhancement leads to the shortening in *T*_1_ relaxation times upon increasing the amount of Yb^3+^ doping. ^207^Pb static NMR measurements show a modest increase in the anisotropy of Pb sites as the concentration of Yb^3+^ was increased. As grinding time was increased, no evident change in the line width of the ^133^Cs signal corresponding to CsPbCl_3_ was observed. There is, however, a decreasing trend in *T*_1_ relaxation, suggesting that the increasing defect density (caused by increasing grinding times) does increase energy exchange between nuclear spins and the lattice. We identified that the *T*_1_ spin relaxation of ^133^Cs was consistently below 35 s and above 13 s for the largest PLQYs observed. However, a thorough comparison of observed PLQYs and measured spin relaxation is not a clear metric for identifying samples with largest PLQYs, given that other factors (dopant clustering/precipitation, presence of nonperovskite phases) affect the optical properties of the synthesized material. In conclusion, ytterbium-doped cesium lead chloride, when synthesized through dry ball-milling, was demonstrated to exhibit the quantum cutting effect across different conditions, namely dopant concentration, excess and depletion of the starting materials, and longer grinding times.

## Supplementary Material



## Notes

Details on sample preparation, reaction schemes, and additional analysis on XRD, SEM/EDX, PL and NMR spectroscopy (PDF)
