# Supplementary material for: Defect Tolerant Quantum Cutting in Mechanosynthesized Ytterbium-Doped Cesium Lead Chloride Perovskites
Source: Chem Mater. 2026 Jan 12;38(2):732–49. doi: 10.1021/acs.chemmater.5c02211 (PMC12854707; doi:10.1021/acs.chemmater.5c02211)
Supplement: Supplementary file 1 [file cm5c02211_si_001.pdf]

Defect Tolerant Quantum Cutting in Mechanosynthesized Ytterbium-doped Cesium Lead Chloride Perovskites

Thiago I. Rubio and Claudia E. Avalos*

Department of Chemistry, New York University, New York, NY 10003, United States

E-mail: claudia.avalos@nyu.edu

Supporting Information Available

Contents

1 Sample preparation and reaction schemes **3**

2 Photoluminescence quantum yield calculation **4**

3 Series A **5**

3.1 pXRD 5

3.2 SEM and EDX 6

3.3 ICP-MS Results 9

3.4 PLQY 9

3.5 Temperature dependence of ^{133}Cs NMR in $CsPbCl_3$ 10

3.6 Effects of Doping on T_2^* 10

3.7 Magnetic field dependence on ^{133}Cs T_1 spin relaxation 12

3.8 Biexponential decay at high Yb concentrations 13
3.9 ^{133}Cs MAS spectra at varying recycle delays 14
3.10 ^{207}Pb solid-state NMR - T_1 relaxation 15
3.11 Linewidths of ^{207}Pb NMR 16
3.12 CSA simultaneous fits of ^{207}Pb static spectra 17

4 Series B **18**
4.1 ^{207}Pb solid-state NMR Spectra 18
4.2 PLQY 19
4.3 Spin relaxation of non-perovskite phases 19
4.4 ^{133}Cs-^{133}Cs NOE Measurements 19

5 Series C **20**
5.1 PLQY 20

6 Series G1 and G2 **20**
6.1 pXRD 20
6.2 Magnetic field dependence on ^{133}Cs T_1 spin relaxation 21
6.3 Low spin-rate ^{133}Cs NMR spectra 22
6.4 Particle size histograms 23
6.5 PLQY 24
6.6 Relationship between different measured properties 25

1 Sample preparation and reaction schemes

Table S1: Molar composition of the starting mixtures for 2.5 mmol Yb^{3+}-doped $CsPbCl_3$

	n(CsCl) (mmol)	n($PbCl_2$) (mmol)	n($YbCl_3$) (mmol)
Series A			
Pristine $CsPbCl_3$	2.50	2.50	0
2.5Yb-A	2.44	2.44	0.063
5Yb-A	2.38	2.38	0.125
10Yb-A	2.25	2.25	0.250
20Yb-A	2.00	2.00	0.500
Series B			
2.5Yb-B	2.50	2.41	0.063
5Yb-B	2.50	2.31	0.125
10Yb-B	2.50	2.13	0.250
20Yb-B	2.50	1.75	0.500
Series C<br>(Varying stoichiometric ratios (5Yb))			
Stoichiometric Cl (5Yb-B)	2.50	2.31	0.125
Excess Cl (5Yb-A)	2.38	2.38	0.125
Higher excess Cl	2.50	2.69	0.125

Series A (2.5 mol% doping):

$$\mathrm{CsCl} + \mathrm{PbCl_2} + 0.025\,\mathrm{YbCl_3} \longrightarrow 2.5\,\mathrm{Yb^{3+}}{:}\mathrm{CsPbCl_{3.08}}$$

As can be seen in the scheme above, each starting mixture includes a stoichiometric excess of chloride ions, respectively 80, 160, 330 and 750 μmol for the 2.5, 5.0, 10 and 20 mol% concentrations of Yb^{3+}. A starting mix that yields a stoichiometric amount of Cl^- was synthesized for each concentration, following the scheme below.

Series B:

$$\mathrm{CsCl} + \left(1 - \tfrac{3}{2}x\right)\mathrm{PbCl_2} + x\,\mathrm{YbCl_3} \longrightarrow x\,\mathrm{Yb^{3+}}{:}\mathrm{CsPb_{(1-\frac{3}{2}x)}Cl_3}$$

This scheme considers the substitution pattern that two Yb^{3+} ions substitute three Pb^{2+} ions and generate one V_{Pb} site. When analyzing the samples however, indication of a secondary phase corresponding to a non-stoichiometric phase was found, hinting that a stoichiometric ratio of CsCl and $PbCl_2$ is necessary to mechanically promote the formation of the $CsPbCl_3$ halide perovskite phase.

In this case, the following stoichiometry was followed: $CsPb_{(1-\frac{3}{2}x)}Yb_xCl_3$, where x was varied from 0.0, 0.025, 0.05, 0.10 and 0.20. We indicate a change of $1-\frac{3}{2}x$ for the lead site given that three lead sites are replaced per two ytterbium atoms with consideration to charge balance. This stoichiometric set of samples was labeled Series B.

2 Photoluminescence quantum yield calculation

PLQYs were calculated using Eq S1.

$$PLQY(QC) = \frac{\left[\frac{\left(A_{sample,NIR}-A_{ref,NIR}\right)\lambda_{em}P_{NIR}}{hcA_{cal,NIR}}\right]}{\left[\frac{\left(A_{sample,UV}-A_{ref,UV}\right)\lambda_{exc}P_{UV}}{hcA_{cal,exc}}\right]} \tag{S1}$$

In Equation S1,

- h is Planck's constant;
- c is the speed of light in vacuum;
- $A_{sample,NIR}$ is the integrated area of the emission band of the sample in the near infrared region;
- $A_{ref,NIR}$ is the integrated area of emission band of the reference matrix in the near infrared region (in this case, the powders are not embedded in any matrix, such as a thin film, so this value was set as zero);
- λ_{em} is the wavelength of the emitted photons (in this case, 985 nm);
- P_{NIR} is the power of the lamp at the emission wavelength;
- $A_{cal,NIR}$ is the integrated area of the spectrum of the lamp around the NIR wavelength collected by the integration sphere with the empty sample holder mounted;

- $A_{sample,UV}$ is the integrated area of the excitation wavelength absorbed by the sample; this is the difference between the total area of the spectrum of the lamp around the excitation wavelength collected using an empty sample holder ($A_{cal,exc}$) and the same radiation band collected by the integration sphere around the excitation wavelength (in this case, 360 nm) with the sample loaded;
- $A_{ref,UV}$ is the integrated area of emission band of the reference matrix in the ultraviolet region (in this case, the powders are not embedded in any matrix, such as a thin film, so this value was set as zero);
- λ_{exc} is the excitation wavelength (in this case, 360 nm);
- P_{UV} is the power of the lamp at the excitation wavelength;
- $A_{cal,exc}$ is the integrated area of the spectrum of the lamp around the UV wavelength collected by the integration sphere with the empty sample holder mounted.

3 Series A

3.1 pXRD

The figure below shows a closer look at pXRD peaks of the orthorhombic phase of $CsPbCl_3$. We note that there is a small shift in the position of this peak as the amount of ytterbium doping is increased. This shift is smaller than previous reports of lattice shrinking in the cubic counterpart of $CsPbCl_3$. Additionally, a broad feature can be identified at 28.5°, and its increase with increasing Yb^{3+} concentration can indicate the precipitation of a distinct phase.

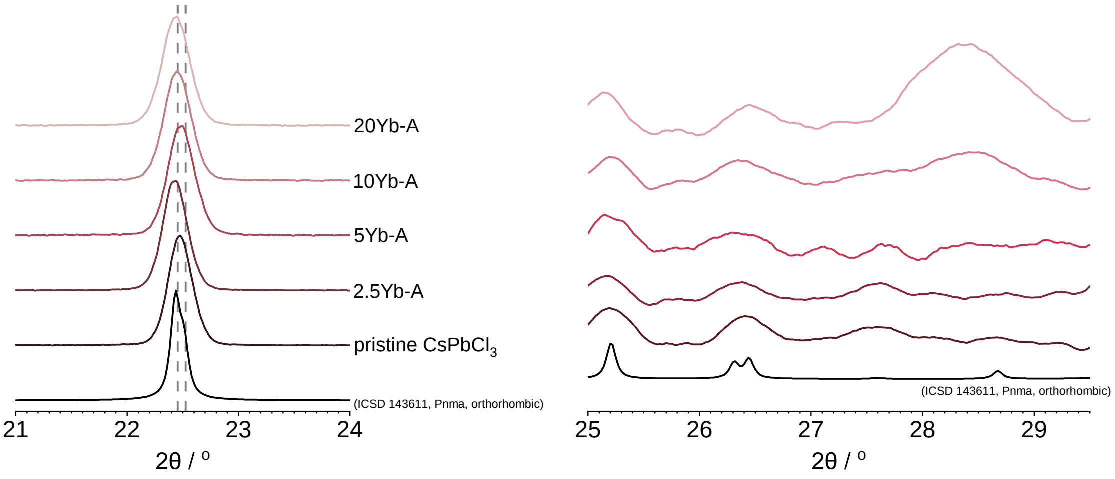

(a) (b)

Figure S1: Closer look at distinct regions of the X-ray diffractograms for series A of samples (a) pXRD shift with increasing Yb^{3+} doping concentration of Series A. (b) A broad feature at 28.5° with increasing intensity is identified at the higher dopant concentrations.

3.2 SEM and EDX

Shown below are SEM and EDX maps of Series A samples doped at 2.5%, 5% and also the pristine case. We observe no aggregation for ytterbium concentrations below 10% in our EDX maps for doped samples.

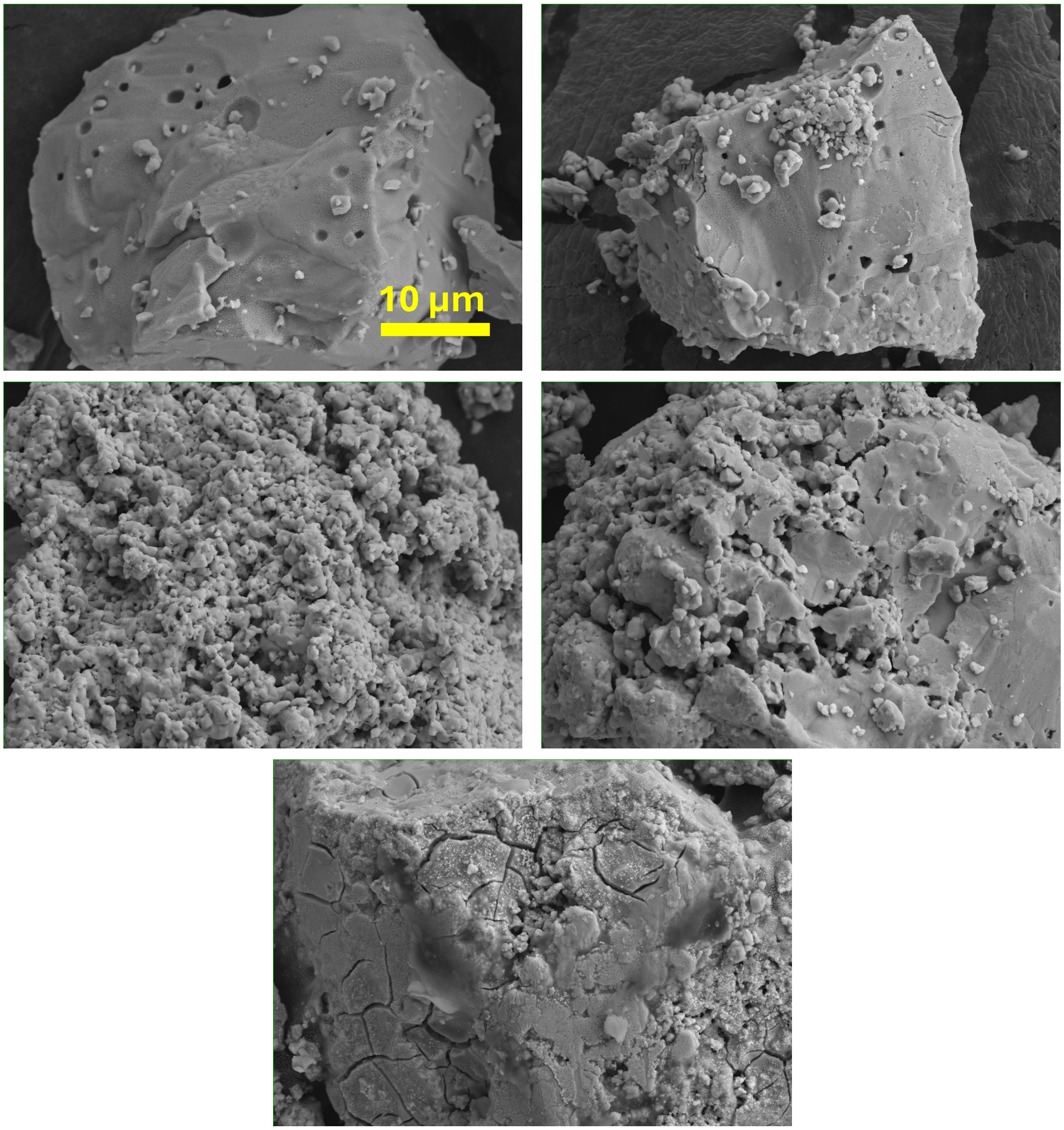

Figure S2: SEM images of mechanosynthesized Yb^{3+} doped $CsPbCl_3$ (Series A). Top left: 0% doping, top right: 2.5% doping. Middle left: 5% doping, middle right: 10% doping, bottom left: 20% doping.

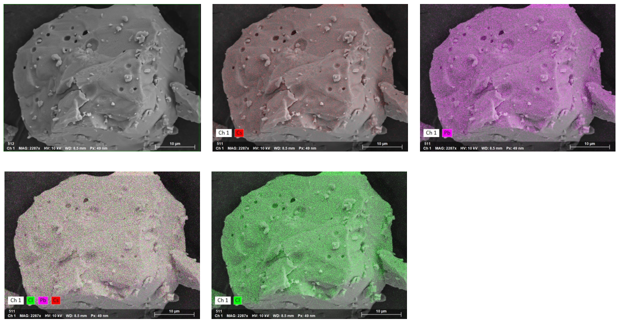

(a)

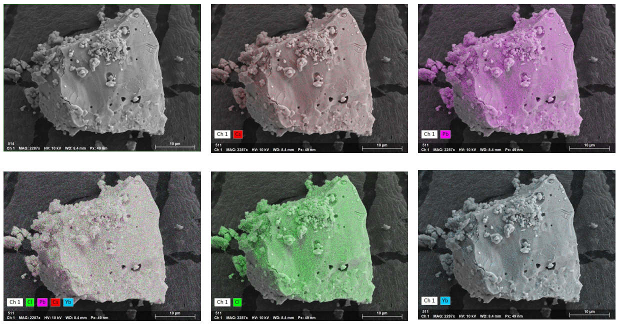

(b)

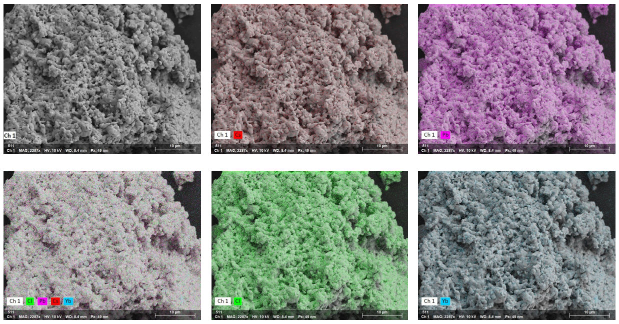

(c)

Figure S3: SEM micrographs and EDX maps for (a) Pristine $CsPbCl_3$, (b) $CsPbCl_3$:2.5Yb^{3+}, and (c) $CsPbCl_3$:5.0Yb^{3+}

3.3 ICP-MS Results

Yb^{3+} ion concentrations in Series A samples were measured in our powders via acid digestion ICP-MS. Approximately 50 mg of each mechanosynthesized sample was digested using hydrofluoric acid and subsequently measured with ICP-MS. All samples were shipped to MSE Supplies LLC in Tucson, Arizona for acid digestion and ICP-MS measurement.

Table S2: Inductive coupled plasma mass spectrometry analysis of Yb in pristine and doped $CsPbCl_3$ (Series A)

Nominal amount of Yb^{3+}	Weight percentage of Yb (%)	Mol percentage of Yb (mol%)
0Yb	0.00	0.00
2.5Yb	1.07	2.73
5.0Yb	2.23	5.65
10Yb	4.39	10.9
20Yb	9.03	21.6

3.4 PLQY

Table S3: Photoluminescence quantum yields (NIR emission) and ^{133}Cs spin-lattice relaxation times fit with a stretched exponential (T_1) of the ytterbium-doped perovskites (Series A)

Sample	PLQY (%)	T_1 (s)
Pristine $CsPbCl_3$	-	206.8 ± 8.1
2.5Yb-A	63.72 ± 2.9	89.6 ± 1.2
5Yb-A	68.55 ± 2.9	30.2 ± 1.9
10Yb-A	72.94 ± 2.9	13.1 ± 2.1
20Yb-A	64.15 ± 2.9	4.2 ± 1.8

3.5 Temperature dependence of ^{133}Cs NMR in $CsPbCl_3$

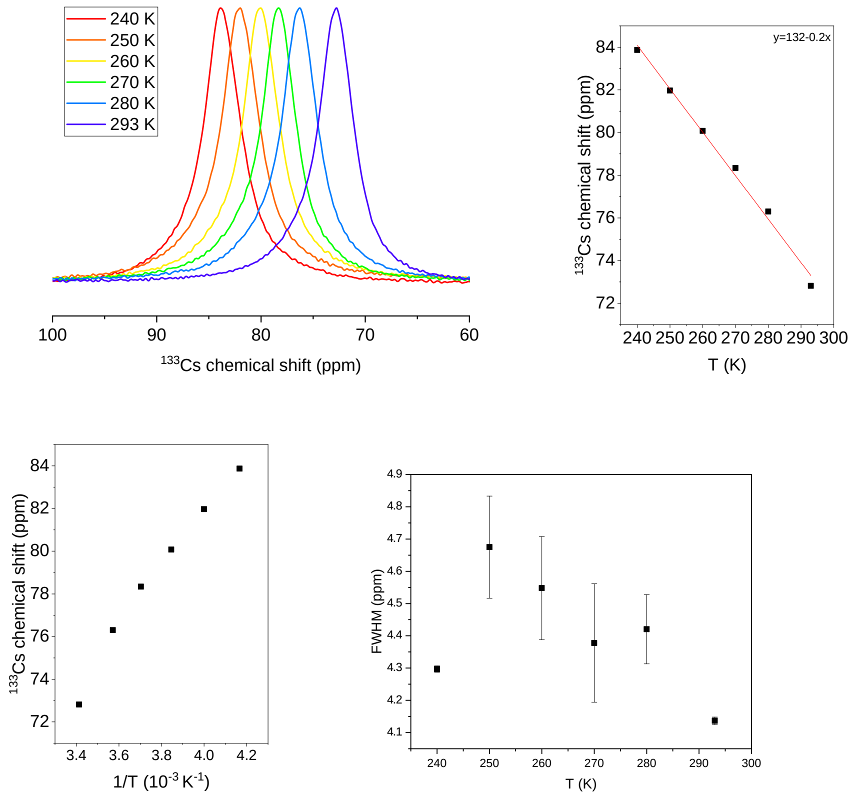

Figure S4: Variation of ^{133}Cs chemical shift in 5Yb:$CsPbCl_3$ (Series A) with temperature. A variation of −0.2 ppm/K is observed for these samples.

3.6 Effects of Doping on T_2^*

If we compare the T_2^* decay of the 20% doped compared to the 5% doped sample we observe a factor of 1.6 decrease in the T_2^* decay. This leads to observable broadening in the collected ^{133}Cs spectra. Free induction decays were fitted using a decaying exponential and ± values are associated with 95% confidence bounds on the fitted value.

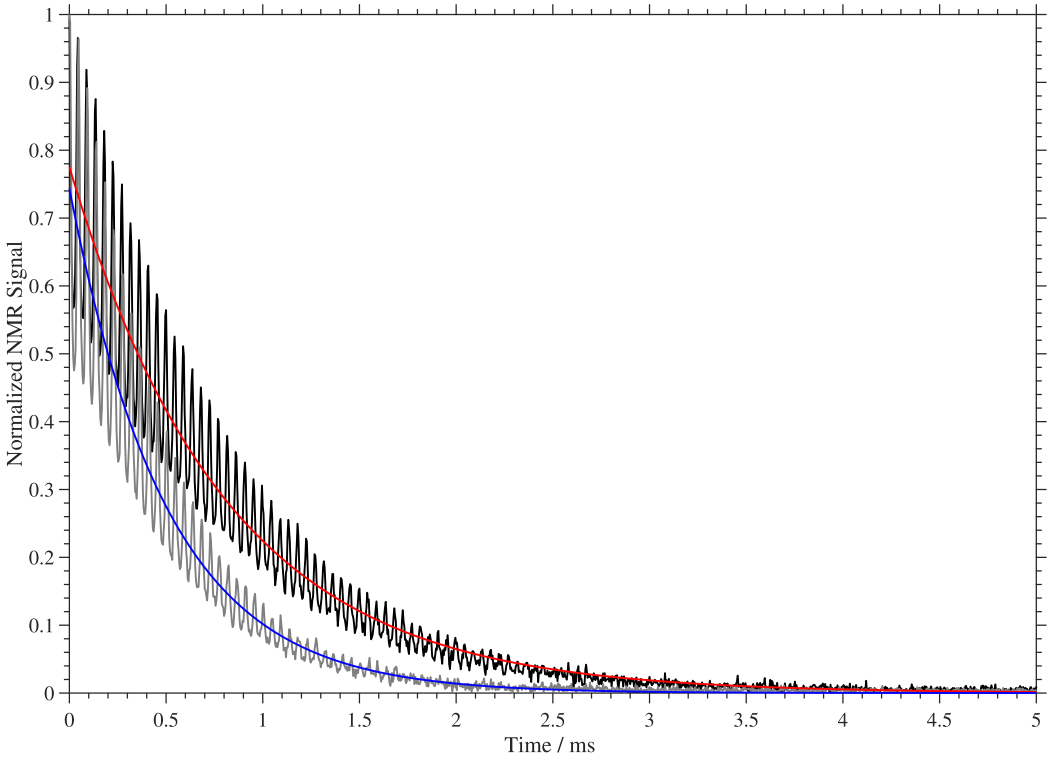

Figure S5: Normalized free induction decay of Series A 20% Yb doped (gray) and 5% Yb doped (black) with corresponding fits yielding a T_2* of 502 ± 4 and 806 ± 5 μs respectively.

3.7 Magnetic field dependence on ^{133}Cs T_1 spin relaxation

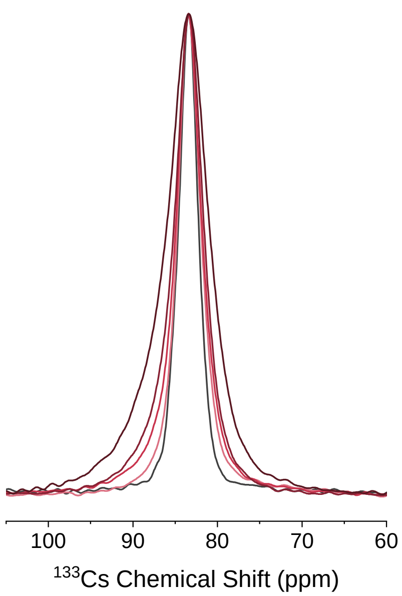

(a) 18.8 T

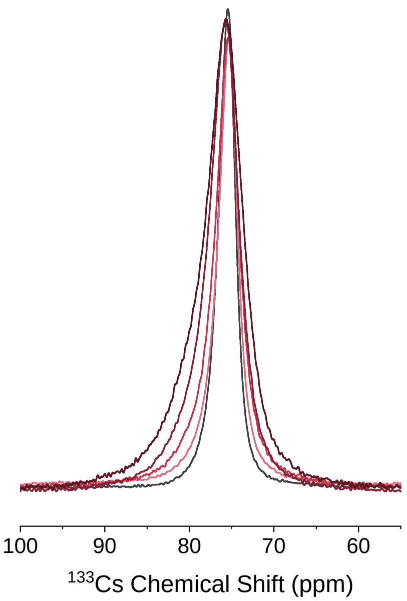

(b) 9.4 T

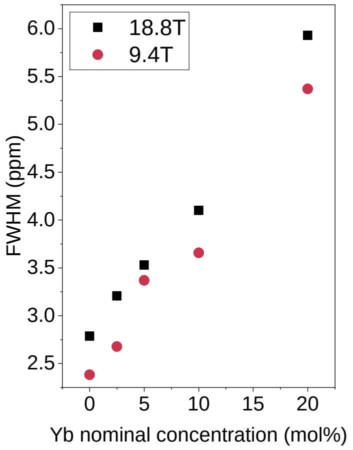

(c)

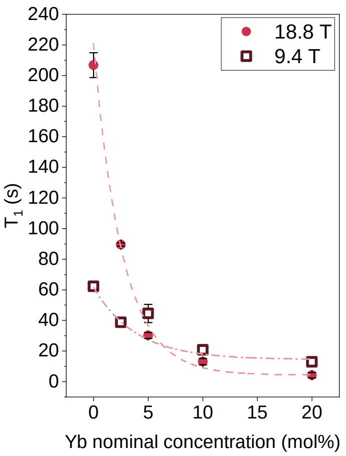

(d)

Figure S6: (a) and (b) ^{133}Cs NMR spectra collected at 18.8 T (240 K) and 9.4 T (270 K) respectively, for the synthesized pristine $CsPbCl_3$ (in dark grey) and Series A of Yb-doped $CsPbCl_3$, varying from 2.5 to 20 mol% (shades of red). The shape of the signal can be described as a skewed-Gaussian with a left-skew extending downfield. In this figure the spectra were corrected for deviations in chemical shift attributed to temperature fluctuations in order to better highlight changes in the lineshape. (c) Full width at half maximum of the central feature in the spectra above, calculated using a Voigt lineshape fit. (d) T_1 measurements obtained at different magnetic fields; the dashed lines correspond to exponential decay fits.

3.8 Biexponential decay at high Yb concentrations

A biexponential fit model was used to describe the spin-lattice relaxation behavior of Series A. We observe that the contribution of the shorter component increases as the ytterbium concentration increases. Both time components decrease as the ytterbium concentration increases.

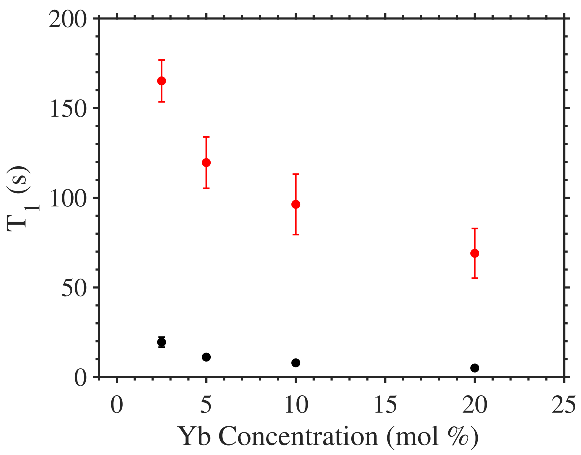

(a)

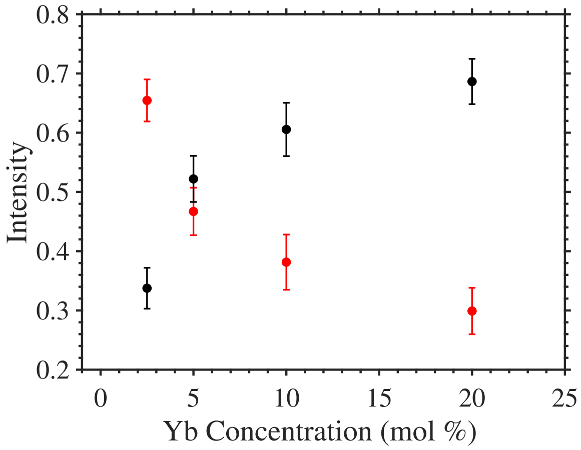

(b)

Figure S7: Biexponential fits for ^{133}Cs saturation recovery data from Series A of samples. (a) T_1 contributions from each component in the fit. (b) Weighting intensity from each component in the biexponential.

3.9 ^{133}Cs MAS spectra at varying recycle delays

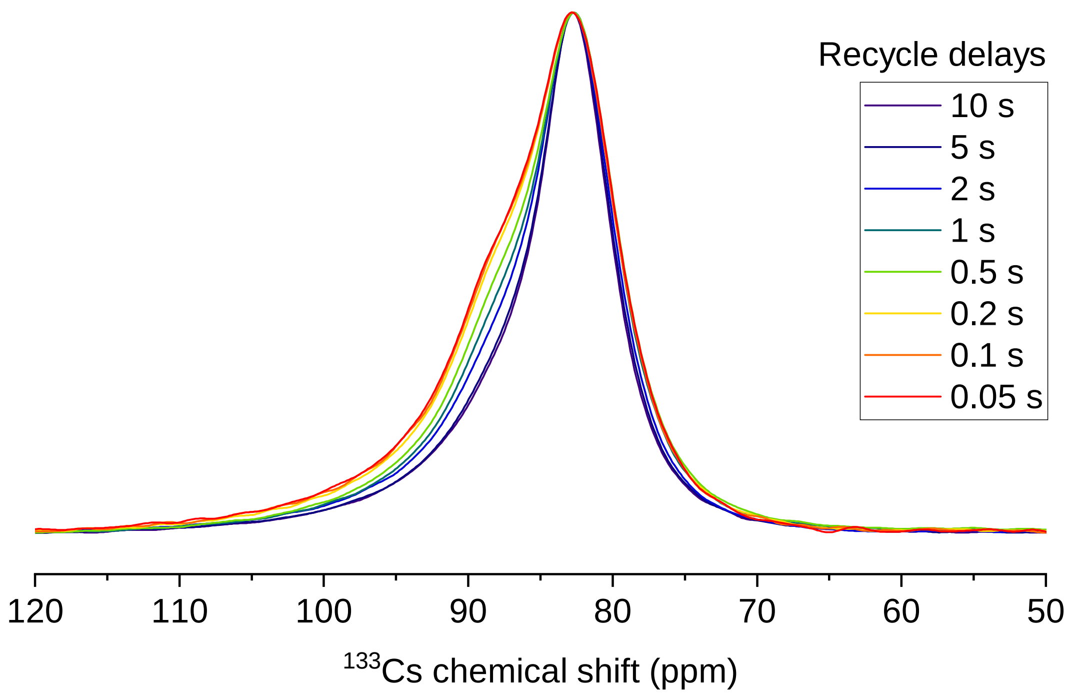

Figure S8: ^{133}Cs NMR for the 20Yb:$CsPbCl_3$ sample from series A measured at different recycle delays. The asymmetric broadening of the lineshape at fast recycling conditions indicates the inclusion of sites with faster relaxation to the distribution of chemical shifts shown, due to pseudocontact shifts. All spectra were measured at 18.8 T, with 22 kHz of spin rate.

3.10 ^{207}Pb solid-state NMR - T_1 relaxation

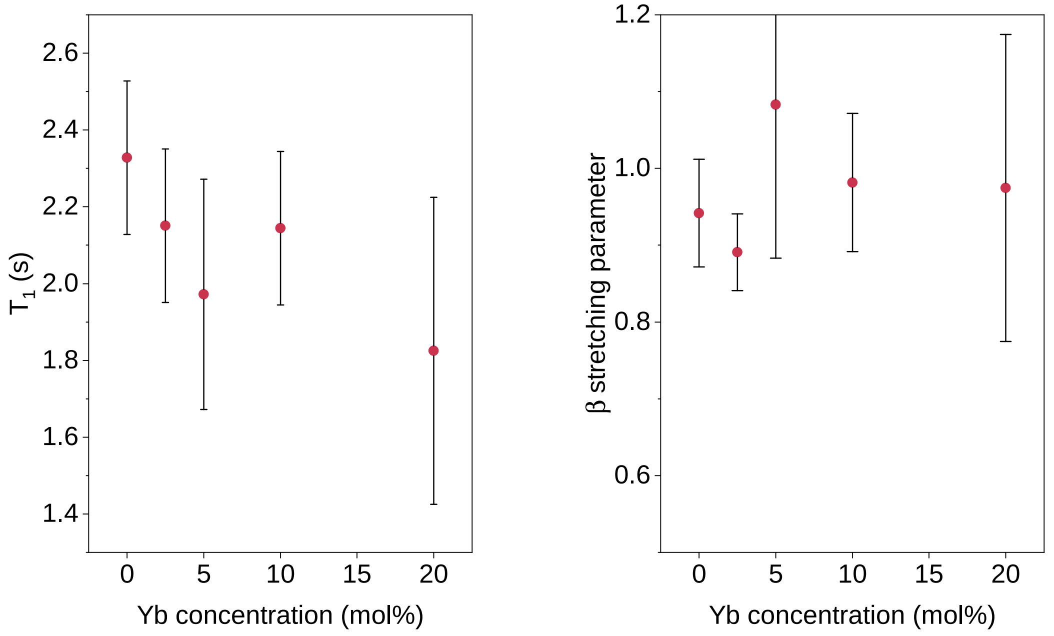

(a) (b)

Figure S9: T_1 (a) and β (b) stretching parameter of ^{207}Pb MAS solid-state NMR spectra (18.8 T, 22 kHz, 240 K) for Series A of samples.

3.11 Linewidths of ^{207}Pb NMR

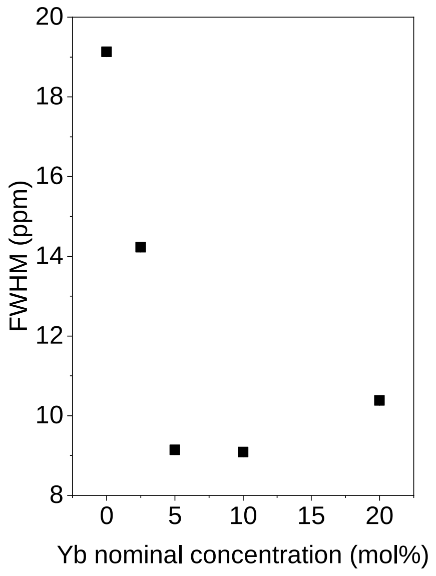

(a)

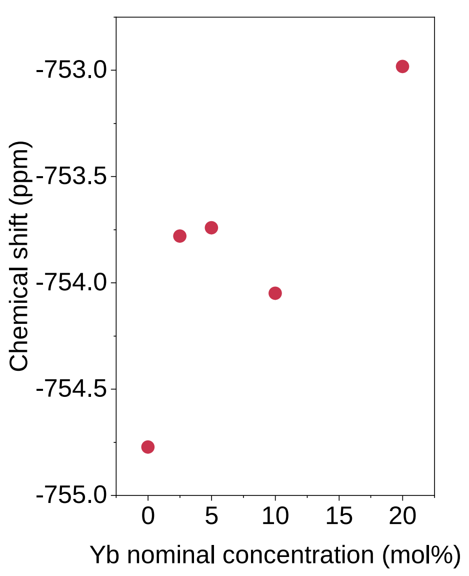

(b)

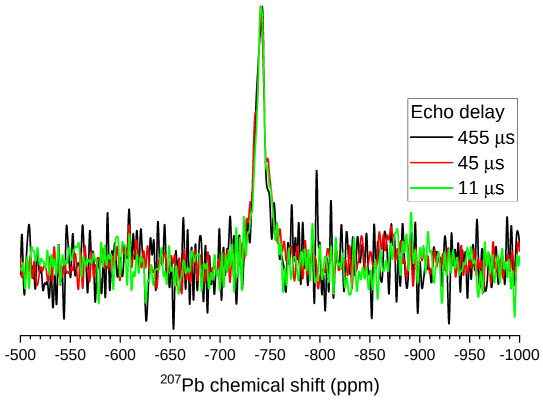

(c)

Figure S10: (a) Full width at half maximum of the ^{207}Pb MAS spectra for series A of samples, calculated using a Voigt lineshape fit (confidence intervals are smaller than the size of the data points).(b) Isotropic chemical shift of the signals as a function of dopant concentration. The change in chemical shift value across the series is attributed to temperature fluctuations. (c) ^{207}Pb spin echo NMR spectra taken using different echo delays τ (spectra taken at 22 kHz, 260 K, 18.8 T).

3.12 CSA simultaneous fits of ^{207}Pb static spectra

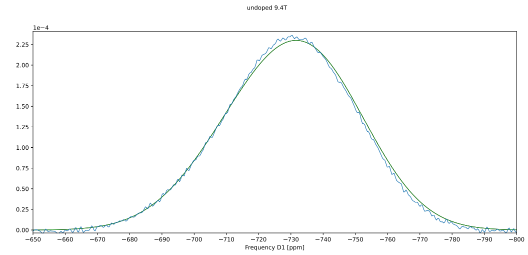

(a)

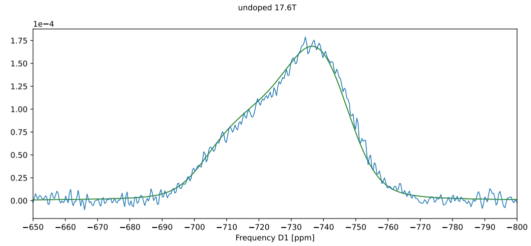

(b)

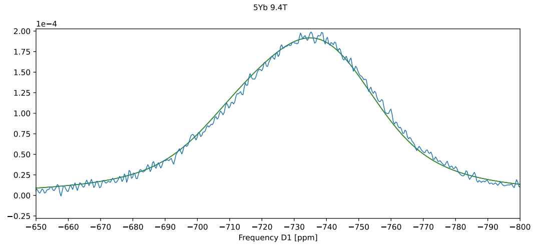

(c)

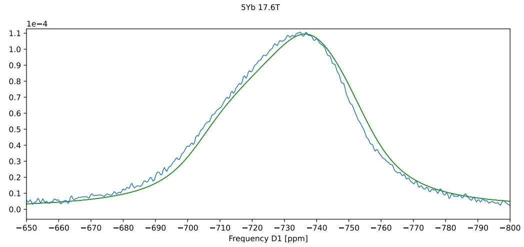

(d)

Figure S11: ^{207}Pb static spectra CSA simultaneous fits. (a) Pristine $CsPbCl_3$ at 9.4 T; (b) Pristine $CsPbCl_3$ at 17.6 T; (c) 5Yb-doped $CsPbCl_3$ at 9.4 T; (d) 5Yb-doped $CsPbCl_3$ at 17.6 T. The blue lines correspond to the experimental data, and the green lines, to the simulated data using the CSA parameters obtained from the fit, which are displayed in the table below.

Table S4: Set of NMR fit parameters obtained for ^{207}Pb solid-state static NMR spectra in the simultanous fit of spectra taken at 9.4 and 17.6 T

**Sample**	δ_{iso} **(ppm)**	Ω **(ppm)**	κ **(ppm)**
Pristine $CsPbCl_3$	-729.8	44.70	-0.5481
5Yb-A	-732.0	50.40	-0.3218

4 Series B

4.1 ^{207}Pb solid-state NMR Spectra

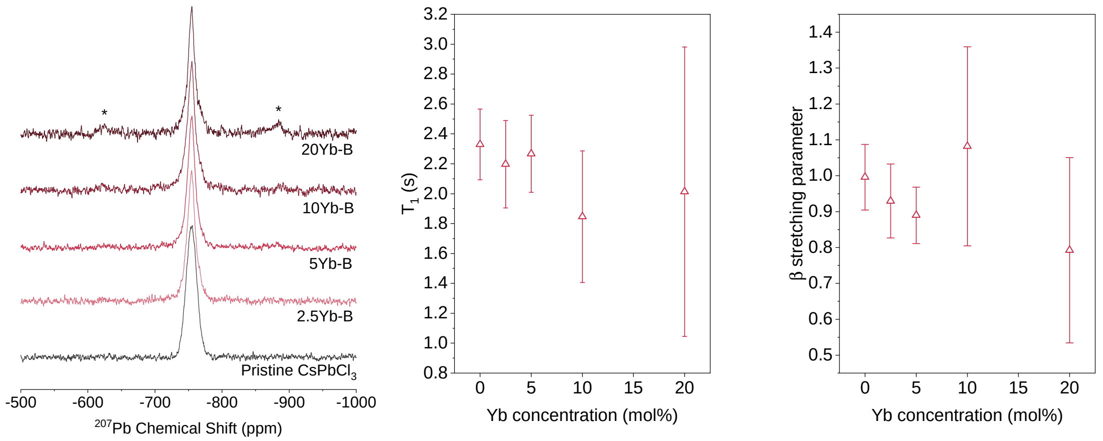

(a) (b) (c)

Figure S12: (a) ^{207}Pb MAS solid-state NMR spectra (18.8 T, 22 kHz, 240 K) for Series B of samples. (b) and (c) show the T_1 relaxation an the β stretching parameter for those samples.

Table S5: Photoluminescence quantum yields for the NIR emission of the ytterbium-doped perovskites, Series B (stoichiometric chloride) and T_1 relaxation of peak at 80 ppm

Sample	PLQY (%)	T_1 (s)
pristine	-	204.0 ± 0.9
2.5Yb-B	59.79 ± 2.9	47.6 ± 0.4
5Yb-B	58.88 ± 2.9	44.5 ± 0.7
10Yb-B	58.86 ± 2.9	36.5 ± 0.5
20Yb-B	50.36 ± 2.9	20.7 ± 0.4

4.2 PLQY

4.3 Spin relaxation of non-perovskite phases

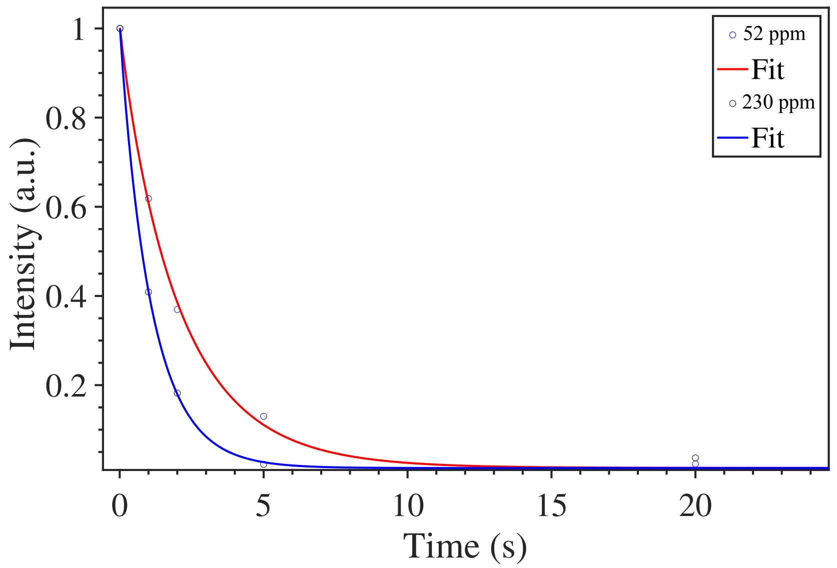

Figure S13: Spin relaxation of ytterbium associated non-perovskite phases in 20% Yb^{3+} doped sample with depleted lead (series B)

4.4 ^{133}Cs-^{133}Cs NOE Measurements

No correlations were observed between distinct Cs sites following 100 μ s of mixing time at 30 kHz MAS. These measurements were repeated again at lower spinning speeds and as in the 30 kHz MAS case, no cross peaks were observed.

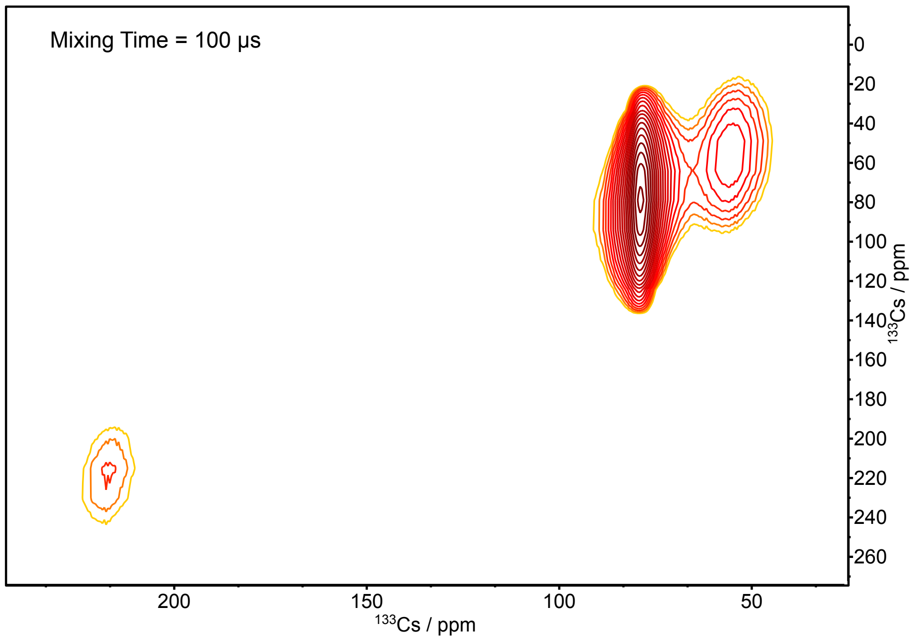

Figure S14: NOESY correlation measurements taken at 30 kHz MAS at 18.8 T with 100 ms of mixing time

5 Series C

5.1 PLQY

Table S6: Photoluminescence quantum yields for the NIR emission of $CsPbCl_3$:5Yb^{3+} at different stoichiometric ratios as well as T_1 relaxation of resonance at 80 ppm

$PbCl_2$:CsCl ratio	PLQY (%)	T_1 (s)
Stoichiometric Cl^-, C_1	55.80 ± 2.9	52.4 ± 0.6
Excess Cl^-, C_2	58.90 ± 2.9	42.0 ± 2.0
Higher excess Cl^-, C_3	60.80 ± 2.9	56.8 ± 1.1

6 Series G1 and G2

6.1 pXRD

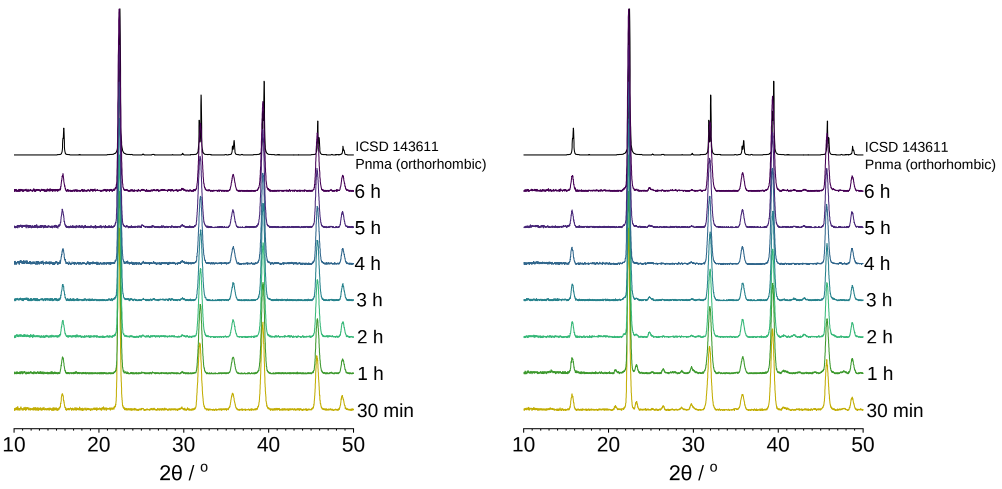

(a) Series G1

(b) Series G2

Figure S15: XRD powder patterns of samples with different ball-milling times, (a) G1, (b) G2.

6.2 Magnetic field dependence on ^{133}Cs T_1 spin relaxation

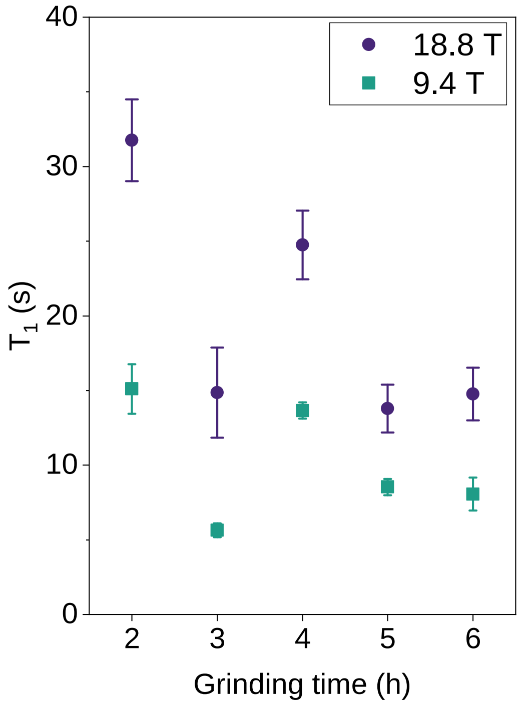

Figure S16: T_1 measurements obtained at different magnetic fields for Series G2 of samples.

6.3 Low spin-rate ^{133}Cs NMR spectra

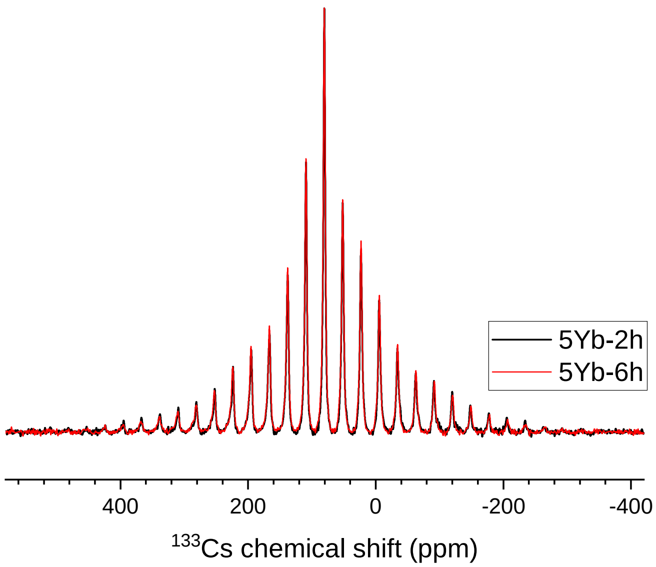

Figure S17: ^{133}Cs solid-state NMR spectra for samples with 2 h and 6 h of grinding time. The relative intensities of the sidebands do not show appreciable changes indicating no significant changes in chemical shift anisotropy of the observed ^{133}Cs site.

6.4 Particle size histograms

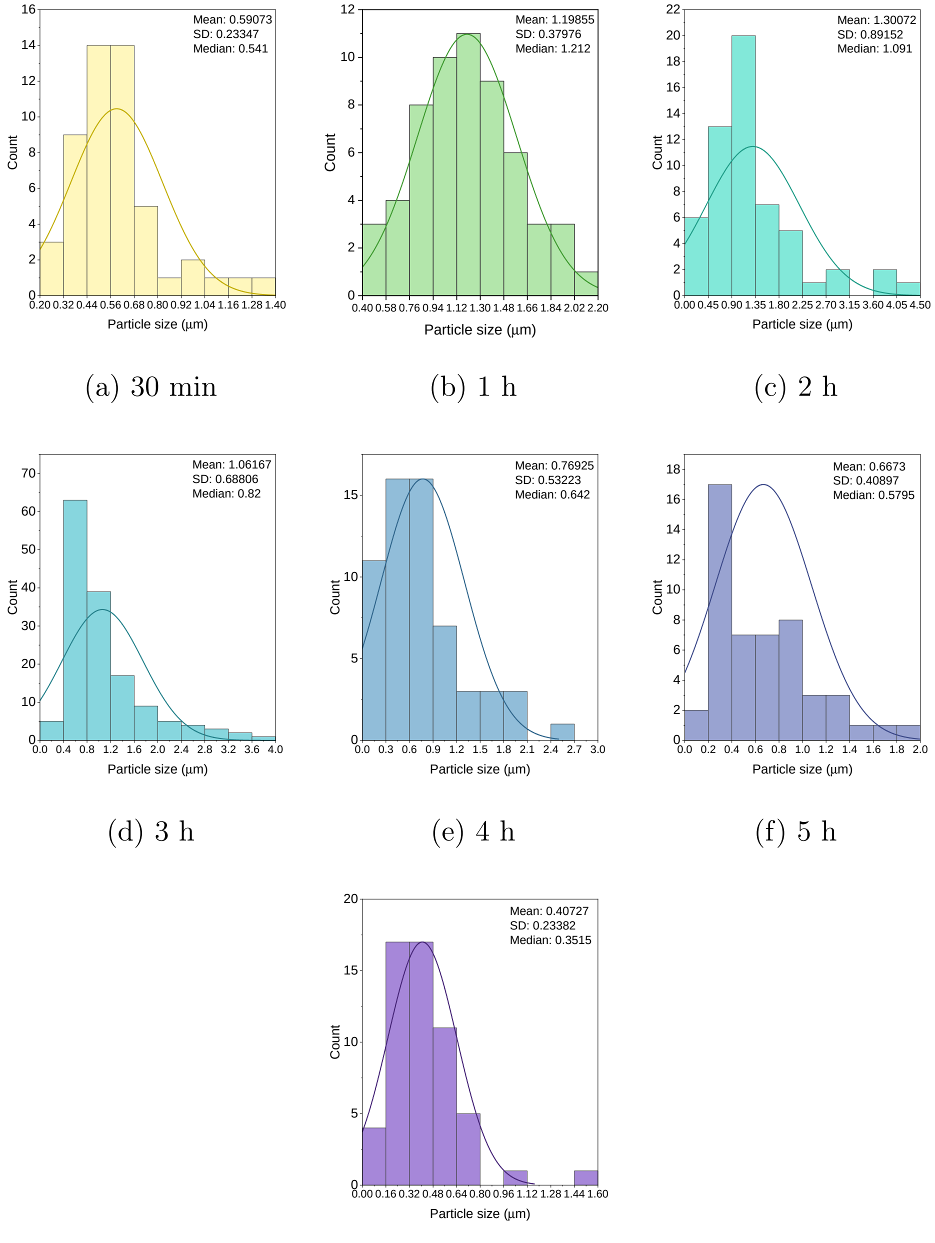

(a) 30 min (b) 1 h (c) 2 h

(d) 3 h (e) 4 h (f) 5 h

(g) 6 h

Figure S18: (a-g) Size distribution histograms constructed from SEM images taken of samples from Series G2.

6.5 PLQY

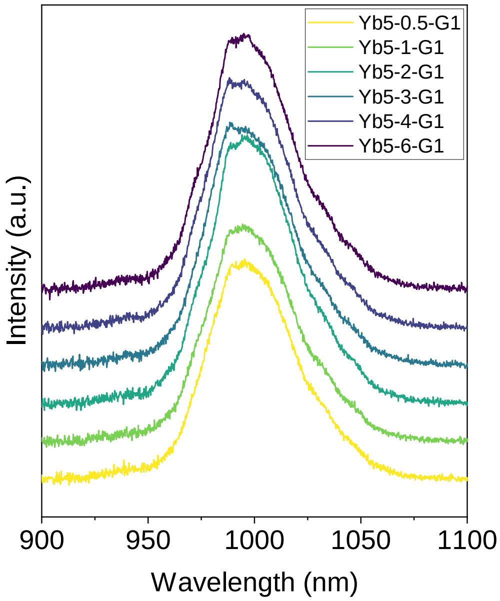

(a)

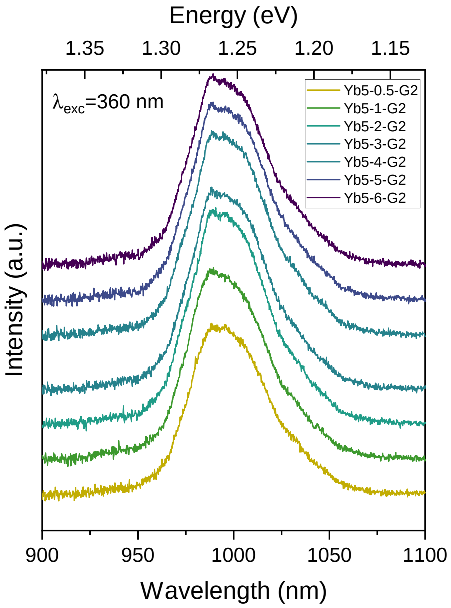

(b)

Figure S19: PL spectra for the samples synthesized with grinding times between 30 min and 6 h ((a) Series G1 and (b) G2).

6.6 Relationship between different measured properties

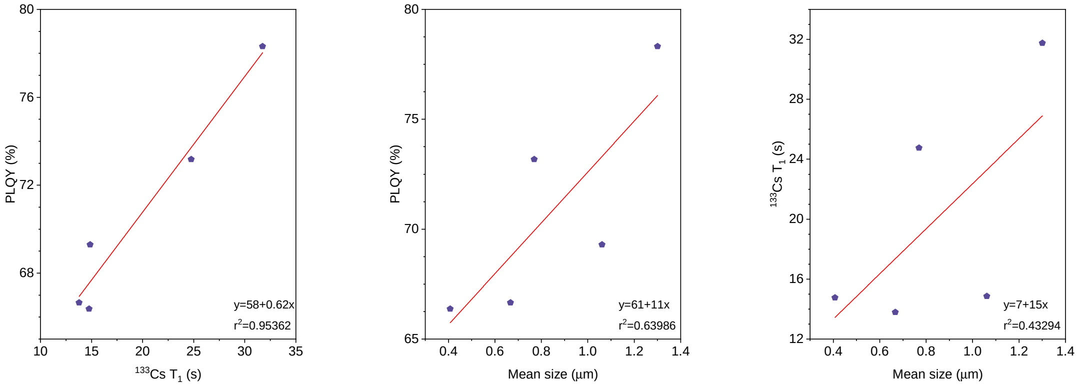

(a) (b) (c)

Figure S20: (a-c) Correlation between ^{133}Cs T_1, mean particle size and PLQY in the phase-pure samples of series G2. Here, we observe that the properties that are more closely correlated are T_1 and PLQY, given a r^2 closer to 1. It is important to highlight that correlation does not imply causation. This means that, when analyzing the effect of grinding time at a fixed concentration, a higher PLQY is not necessarily caused by a longer longitudinal relaxation behavior of ^{133}Cs.
